# Bee Venom for the Treatment of Parkinson Disease – A Randomized Controlled Clinical Trial

**DOI:** 10.1371/journal.pone.0158235

**Published:** 2016-07-12

**Authors:** Andreas Hartmann, Julia Müllner, Niklaus Meier, Helke Hesekamp, Priscilla van Meerbeeck, Marie-Odile Habert, Aurélie Kas, Marie-Laure Tanguy, Merry Mazmanian, Hervé Oya, Nissen Abuaf, Hafida Gaouar, Sabrina Salhi, Fanny Charbonnier-Beaupel, Marie-Hélène Fievet, Damien Galanaud, Sophie Arguillere, Emmanuel Roze, Bertrand Degos, David Grabli, Lucette Lacomblez, Cécile Hubsch, Marie Vidailhet, Anne-Marie Bonnet, Jean-Christophe Corvol, Michael Schüpbach

**Affiliations:** 1 Assistance Publique Hôpitaux de Paris (APHP), UPMC, INSERM, ICM, Centre d’Investigation Clinique Pitié Neurosciences, CIC-1422, Département des Maladies du Système Nerveux, Hôpital Pitié-Salpêtrière, Paris, France; 2 Assistance Publique Hôpitaux de Paris (APHP), Service de Médecine Nucléaire, Hôpital Pitié-Salpêtrière, Paris, France; 3 Assistance Publique Hôpitaux de Paris (APHP), Unité de Recherche Clinique, Hôpital Pitié-Salpêtrière, Paris, France; 4 Assistance Publique Hôpitaux de Paris (APHP), Service de Dermatologie et d’Allérgie, Hôpital Tenon, Paris, France; 5 Assistance Publique Hôpitaux de Paris (APHP), Agence Générale des Equipements et Produits de Santé, Paris, France; 6 Assistance Publique Hôpitaux de Paris (APHP), Pharmacie, Secteur Essais Cliniques et Dispensation des Médicaments aux Patients Externes, Hôpital Pitié-Salpêtrière, Paris, France; 7 Assistance Publique Hôpitaux de Paris (APHP), Service de Neuroradiologie, Hôpital Pitié-Salpêtrière, Paris, France; Institute for Clinical Epidemiology and Applied Biometry, GERMANY

## Abstract

In the present study, we examined the potential symptomatic and/or disease-modifying effects of monthly bee venom injections compared to placebo in moderatly affected Parkinson disease patients. We conducted a prospective, randomized double-blind study in 40 Parkinson disease patients at Hoehn & Yahr stages 1.5 to 3 who were either assigned to monthly bee venom injections or equivalent volumes of saline (treatment/placebo group: n = 20/20). The primary objective of this study was to assess a potential symptomatic effect of s.c. bee venom injections (100 μg) compared to placebo 11 months after initiation of therapy on United Parkinson’s Disease Rating Scale (UPDRS) III scores in the « off » condition pre-and post-injection at a 60 minute interval. Secondary objectives included the evolution of UPDRS III scores over the study period and [123I]-FP-CIT scans to evaluate disease progression. Finally, safety was assessed by monitoring specific IgE against bee venom and skin tests when necessary. After an 11 month period of monthly administration, bee venom did not significantly decrease UPDRS III scores in the « off » condition. Also, UPDRS III scores over the study course, and nuclear imaging, did not differ significantly between treatment groups. Four patients were excluded during the trial due to positive skin tests but no systemic allergic reaction was recorded. After an initial increase, specific IgE against bee venom decreased in all patients completing the trial. This study did not evidence any clear symptomatic or disease-modifying effects of monthly bee venom injections over an 11 month period compared to placebo using a standard bee venom allergy desensitization protocol in Parkinson disease patients. However, bee venom administration appeared safe in non-allergic subjects. Thus, we suggest that higher administration frequency and possibly higher individual doses of bee venom may reveal its potency in treating Parkinson disease.

**Trial Registration:**

ClinicalTrials.gov NCT01341431

## Introduction

The cardinal motor symptoms of Parkinson disease (PD), akinesia, rigidity and rest tremor, are due to degeneration of dopaminergic neurons in the substantia nigra pars compacta (SNpc) with subsequent depletion of dopamine in the striatum. This, in turn, modifies the activity of basal ganglia output structures [1]. Therefore, symptomatic therapies in PD aim at either repleting dopamine or normalising basal ganglia activity, for instance by high frequency deep brain stimulation (DBS) of the globus pallidus internus (GPi) or the subthalamic nucleus (STN). The holy grail, however, remains the development of neuroprotective strategies to slow degeneration of nigral dopaminergic neurons and thus disease progression, at least with regard to the motor triad. We propose that bee venom may exert both symptomatic and neuroprotective effects in PD.

Regarding potential neuroprotective effects of bee venom, hyperpolarization of midbrain dopaminergic neurons resulting from blockade of Ca^2+^-activated small conductance K^+^ (SK) channels by the bee venom toxin apamin partially rescues dopaminergic neurons from their spontaneous demise in dissociated mesencephalic cultures [2]. Apamin is the only polypeptide neurotoxin contained in bee venom to pass the blood-brain-barrier when injected peripherally (18 aa peptide) and irreversibly blocks SK channels [3]. These channels (subtypes 1–3) are present in various neuronal populations throughout the central nervous system and play a major role in the control of the switch between tonic and burst firing in physiological conditions [4]. SK3 channels can be detected on nigral dopaminergic neurons. Taken together, this suggests that SK channel blockade of dopaminergic neurons not only controls their firing pattern but ultimately, their survival [5].

Regarding the hypothesis that bee venom might exert acute, symptomatic effects on PD motor symptoms, we suggest that these are not be due to striatal dopamine release only. In 1-methyl-4-phenyl-1,2,3,6-tetrahydropyridine-treated (MPTP) mice [6] and 6-hydroxydopamine-lesioned rats [7], bee venom and/or apamin raise striatal dopamine levels but the kinetics of this increase are likely long-term as well as short-term. Therefore, a complementary option is that blockade of SK channels downstream of the dopaminergic nigrostriatal system, i.e. on basal ganglia relay or output nuclei may mediate motor effects. Indeed, SK2 receptors are present on GABAergic substantia nigra pars reticulata (SNpr) and glutamatergic subthalamic nucleus (STN) neurons [4], and bee venom restores the balance between inhibitory and excitatory influence exerted by the striatum and the STN on SNpr cells following dopaminergic transmission interruption by neuroleptics injection, thereby normalising basal ganglia output activity [8]. This effect is almost identical with that observed following DBS of the STN in rats [9].

Several recent studies have adressed the potential use of bee venom therapy in PD models and PD patients [6,7,8,10,11,12,13]. Based on these findings, we conducted a monocentric double-blind, randomized controlled pilot study to evaluate the potential symptomatic and disease-modifying effects of monthly bee venom injections, as used in classic desensitization protocols against bee venom allergy, in moderately affected PD patients.

## Methods

### Patients

All clinical investigations were performed in accordance with the tenets of the Declaration of Helsinki. All patients provided their written informed consent to participation. Our local institutional review board (Pitié-Salpêtrière Hospital, Paris/France) approved the aims and procedures of the study (national reference number: 2009-016702-16; ClinicalTrials.gov reference: NCT01341431). For the CONSORT checklist, please see S1 Fig.

Patients included suffered from PD as defined by the Parkinson’s Disease Society Brain Bank [14]. They were over age 40 (to exclude young-onset PD forms), at Hoehn and Yahr stages 1,5–3 during « off » periods, had a pathological [123I]-FP-CIT, an MRI excluding secondary or atypical forms of parkinsonism and had a negative skin test (ST) to bee venom. Recruitment took place between April 2011 and June 2012 at the Department of Neurology, Pitié-Salpêtrière Hospital, Paris/France.

Exclusion criteria were a Hoehn & Yahr stage <1,5 or >3, a secondary or atypical parkinsonian syndrome (verified clinically and by MRI), neuroleptic treatment within the last six months except for domperidone, renal or hepatic insufficiency, abnormal ECG, normal [123I]-FP-CIT, pregnancy or absence of contraception if in procreating age, non-treated major depression or other severe psychiatric disorder according to DSM-IV-TR or cognitive impairment (Mini Mental State (MMS) < 24/30). In addition, patients were not included if they had a known allergy against bee venom or a contraindication against the use of pharmaceutical-grade bee venom (Alyostal®, Stallergenes, Antony, France), if ST against bee venom was positive or if specific IgE antibodies against bee venom could be detected (>0.1 kU/L) at the screening visit.

### Study design

We performed a randomized, double-blind, placebo-controlled, parallel-group single-center trial. Treatment was allocated according to a computer-generated randomization list in a 1:1 ratio. Randomization was performed via a central web-based system (http://randoweb.aphp.fr). Patients were randomized into two groups and received either bee venom 100 μg (Alyostal®) or placebo s.c. once a month over an 11 month periods (V2-V13). Depending on the result of randomization and to maintain the blind, the pharmacy of the hospital prepared kits containing either a syringe of bee venom (Alyostal® 100 μg in 1 mL of NaCl 0.9%) or a syringe of placebo (NaCl 0.9%, 1 mL) that were identical in appearance. Then, these kits were labeled with indications of research and patient identity, and were dispensed to the medical team for each visit of administration.

For the conduction of ST, Alyostal® was diluted in sterile saline solution containing 0.4% phenol at a concentration of 0.1 μg/mL. Intradermal ST was done on the volar surface of the forearm. Through a 25-gauge hypodermic needle, beginning with the highest dilution of bee venom, 0.05 mL were injected. Prick ST with histamine (10 mg/mL) and phenol-saline diluent solution were used as positive and negative controls, respectively. The reactions were read after 20 minutes. The cut-off value for a positive intradermal ST was a wheal diameter ≥ 6 mm associated with a flare ≥ 10 mm and at least 70% of the histamine control [15] as well as a negative response to the control phenol-saline solution.

Serum IgE and IgG4 antibody measurements were performed using a fluorescence-immunoassay (CAP-FEIA) with commercially available total bee venom Immunocap and a Phadia Unicap100 instrument, according to the manufacturer’s instructions (Thermo Fisher Scientific, Waltham, MA). Isotype of the antibodies, IgE or IgG4, was determined with ImmunoCAP specific IgE conjugate and Immunocap specific IgG4 conjugate. Results were calculated in kU/L for IgE antibodies and in mg/L for IgG4 antibodies. Limits of detection for IgE was 0.1 kU/L and for IgG4 0.007 mg/L.

Patients were seen monthly after the pre-screening (V0) and selection (V1) visits. Subsequently, bee venom or placebo injections were performed over an 11 month period (V2-13) and study participation ended with a final follow-up visit (V14). The United Parkinson’s Disease Rating Scale (UPDRS) [16] was performed in the « off » condition, before the first morning dose, after withdrawal of their levodopa treatment the evening before the visit. In case of treatment with dopamine agonists, rasagiline or amantadine, treatments were stopped at least five drug half-lives before evaluation. If necessary, equivalent doses of levodopa were administered in the meantime to minimize patient discomfort.

We also used a segmental rating scale [17] which is likely more sensitive to change than the UPDRS III. The segmental score was calculated as follows:

- The *action and kinetic tremor score* was the sum of all items for action and kinetic tremor (but not rest tremor)
- For rest tremor rating always the worst tremor was rated. For rigidity rating, always the least rigidity at rest was rated.
- The *tremor severity* score was the sum of all rest tremor items.
- The *rigidity severity* score was the sum of all rigidity items.
- The *composite rigidity and tremor severity* score was the sum of the tremor severity score and the rigidity severity score.

### Brain imaging

All patients underwent a brain MRI on a 3T MR unit (Siemens, Erlangen, Germany). Sequences performed included a 3DT1 (sagittal plane acquisition, gradient echo sequence with inversion recovery, 1 mm isotropic voxel), a 3DT2 (sagittal plane acquisition, fast spin echo sequence, fast spin echo acquisition, 1 mm isotropic voxel) and an axial FLAIR (fluid attenuated inversion recovery) sequence. Total acquisition time was 20 minutes. Images were analyzed by an experienced neuroradiologist (DG).

SPECT studies were performed in all subjects using a triple-head camera equipped with low energy, high resolution parallel hole collimators (Irix, Philips), 3 hours after injection of 185 MBq (range 165–188 MBq) of [123I]-FP-CIT. Thyroid uptake was blocked before the scan by administration of potassium perchlorate (400 mg orally). One hundred and twenty projections were acquired for 30 minutes in a 128 x 128 matrix. Projections were reconstructed using an iterative algorithm, post-filtered (low pass filter: order = 4, cut-off frequency = 0.35 cm-1), then corrected for attenuation using the Chang method (μ = 0.12 cm^-1^) [18]. To ensure reproducibility between scans, patients were injected with the same dose (± 5%), with the same interval between injection and acquisition, and same rotation radius to ensure similar resolution.

All reconstructed volumes were converted from DICOM to NIFTI format. Parametric images of binding potential (BP) were computed from the reconstructed [123I]-FP-CIT volumes using the Anatomist and Brainvisa software packages (http://brainvisa.info/). For each FP-CIT study, a volume of interest was drawn within the occipital cortex to obtain non-specific activity, and BP was calculated in each voxel using the following formula: (mean voxel activity / mean occipital activity) minus 1. The resulting parametric volumes were then spatially normalized to the Montreal Neurological Institute (MNI) space with the Statistical Parametric Mapping software (SPM8, Welcome Department of Cognitive Neurology, University College, London) using a custom [123I]-FP-CIT template [19]. Next, BP was estimated for each parametric image in a standard set of specific volumes of interest (VOI), i.e. right and left caudate nuclei, anterior and posterior putamens, and whole striatum. These VOI were manually delineated on the MNI single subject MRI. This method has been previously validated [19].

### Safety assessment

The patients’ general health status (as reflected by body weight, systolic/ diastolic arterial blood pressures, heart rate, electrocardiogram, and standard blood biochemistry profile) was assessed every month. IgE testing was performed every two months. In case of significant rise of antibodies, STs were repeated.

### Objectives

The primary objective of this study was to assess a potential symptomatic effect of s.c. bee venom injections (100 μg) compared to placebo 11 months after initiation of therapy (at V13) on UPDRS III scores in the « off » condition pre-and post-injection at a 60 minute interval. This choice was based on two hypotheses: (i) that a potential symptomatic effect would build up over the study period; and (ii) that this effect would manifest itself 30–60 minutes, at latest, after injection of bee venom.

Four secondary objectives were pre-specified as follows:

- Study the evolution of the symptomatic effect of bee venom along time (V2-13) as compared to placebo on UPDRS III scores in the « off » condition at a 60 minute interval to verify whether an effect might build up or vanish over time.
- Study the potential effect of monthly bee venom injections compared to placebo on disease progression in the « off » condition by assessing UPDRS III scores between V2 and V13. Dopaminergic treatment modifications over the same period were used as a concomitant source of information on disease progression.
- Study the potential effect of monthly bee venom injections compared to placebo on disease progression by dopaminergic denervation as assessed by [123I]-FP-CIT between V2 and V13.
- Study the potential effect of monthly bee venom injections compared to placebo on motor fluctuations by assessing UPDRS IV scores between V2 and V13.

### Statistical analysis

Sample size was calculated assuming a mean of UPDRS III score before injection at visit 13 equal to 28 points, an improvement of 20% (i.e 5.6 points) after injection at visit 13 in the active group without change in the placebo group and a standard deviation of 5.6 points in each arm. To provide the study with a power of 80%, 40 patients had to be randomized. The sample size was determined based on a 2-sided type I error rate of 5% using the Mann-Whitney test.

The primary efficacy analysis was performed using the modified intent-to-treat (mITT) population (defined as all randomized subjects who took at least one dose of study medication). The primary criterion was missing for five patients in the bee venom arm (four positive ST during the study period and one patient withdrawal). According to the protocol, these patients were considered as failures with a variation of UPDRS III score after V13 equal to zero (no improvement). The change in UPDRS III score between before and after injection at V13 was compared with the Mann-Whitney test between the two groups. Change from baseline to V13 in UPDRS III score after each monthly injection was studied with a linear mixed model for repeated measures included terms for treatment group, time and time by treatment interaction. Change from baseline in UPDRS I, II and IV and in the segmental rating scale were also analyzed with a linear mixed model for repeated data. Changes from baseline in Parkinson’s disease questionnaire (PDQ)-39 summary index and in dopamine transporter binding potential as assessed by [123I]-FP-CIT was compared between arms using the Mann-Whitney test. The alpha level was set at 0.05 (two-sided). All analyses were performed with the SAS software version 9.2 (SAS Institute, Cary, NC).

## Results

### Patient characteristics at baseline

**Fig 1:**
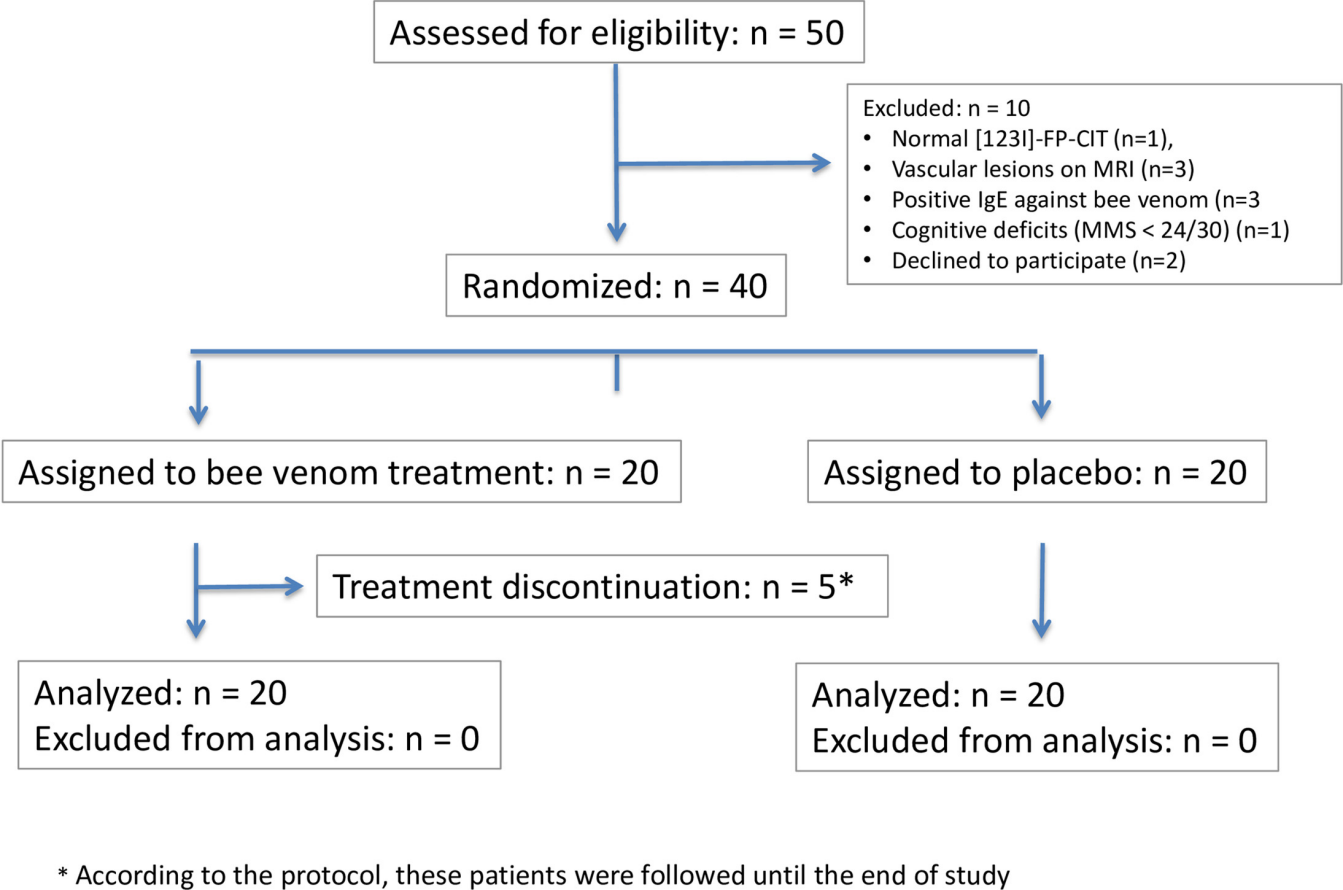
Study Flowchart.

Fifty patients diagnosed with PD were screened for participation in the trial. One subject had a normal [123I]-FP-CIT, three had vascular lesions on MRI, three had positive IgE against bee venom, one had cognitive deficits (MMS < 24/30) and two decided to not pursue the study before randomization. Eventually, 40 patients were included into the study (Fig 1). Even if patients who discontinued treatment and for which the primary outcome could not be collected were excluded from the analysis, the difference between groups remained non-significant (p = 0.68).

Twenty patients were included in the placebo arm (12 male, 8 female) and 20 patients in the treatment (bee venom) arm (8 male, 12 female). Baseline demographic characteristics were similar in both groups (Table 1). Although not significantly different between groups, it is worth noting that patients in the bee venom group has lower UPDRS III scores and lower levodopa-equivalent daily dose (LED) than their placebo counterparts, yet scored higher on the UPDRS IV subscale.

**Table 1:** Baseline characteristics in the placebo / bee venom groups.

	Placebo (Median ; Interquartile range)	Bee venom (Median ; Interquartile range)
Age (years)	63.3 ; 8	60.3 ; 15
Time since diagnosis (years)	5.6 ; 4	5.9 ; 4.4
Time since first symptoms (years)	6.3 ; 5.1	6.2 ; 5
BREF	17 ; 0.5	17 ; 1.5
MMS	30 ; 2	29 ; 2
UPDRS I	1 ; 1	1 ; 2
UPDRS II	10.5 ; 7	9.5 ; 5
UPDRS III (off)	29 ; 16	25 ; 11
UPDRS IV	3; 2.5	3.5; 3.5
Schwab and England scores		
- 70	2 (10%)	0
- 80	13 (65%)	9 (45%)
- 90	4 (20%)	9 (45%)
- 100	1 (5%)	2 (10%)
Hoehn and Yahr stages		
- 2	7 (35%)	6 (30%)
- 2.5	11 (55%)	14 (70%)
- 3	2 (10%)	0
PDQ-39 –summary index	22.6; 20	24.7 ; 12.7
LED	512 ; 450	391 ; 306

### Primary outcome

**Fig 2:**
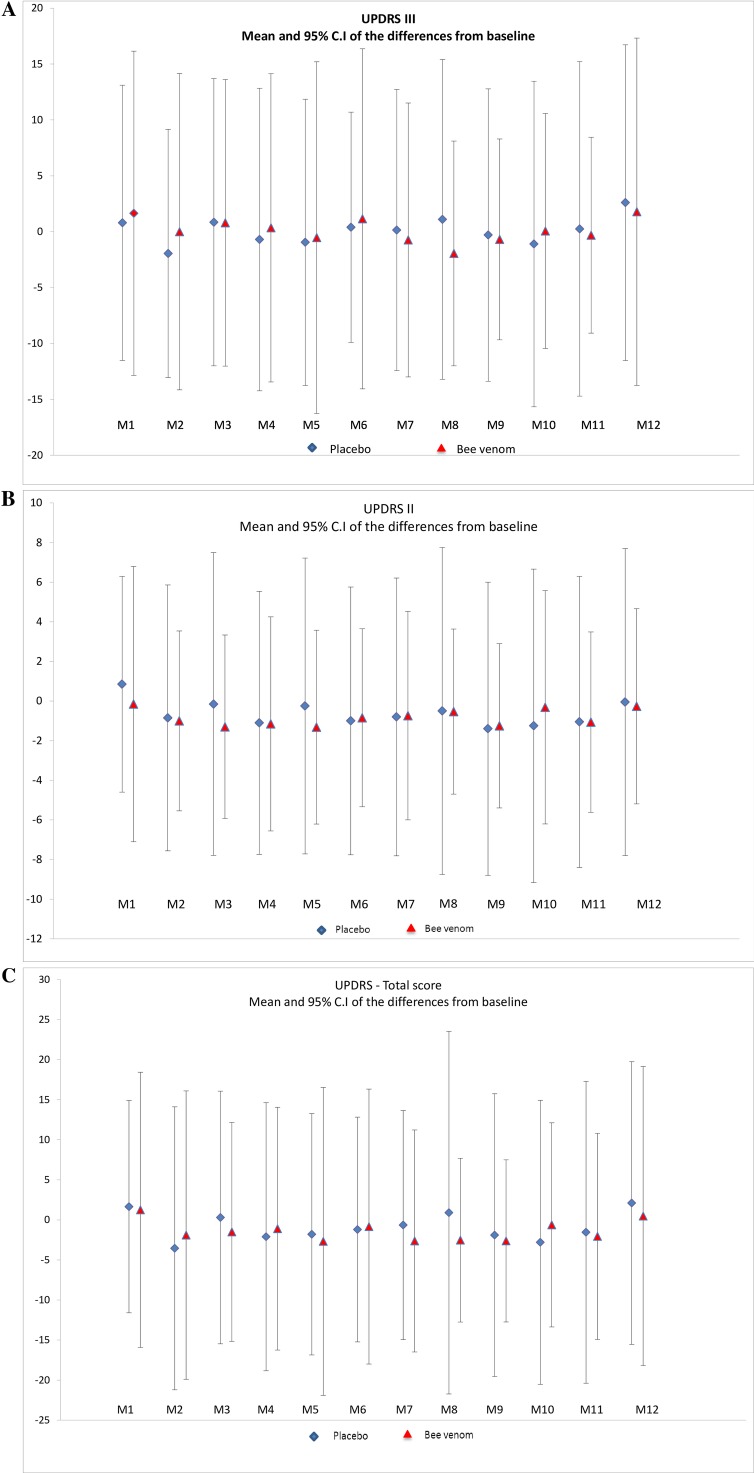
Evolution of the differences of UPDRS III (A), II (B) and total scores (C) with baseline over the 11 month study period in the placebo and bee venom groups.

At V13, UPDRS III scores decreased both after placebo and bee venom injections (Table 2) and the differences between the two groups were non-significant. With regard to disease progression, UPDRS III scores in the “off” state in both groups did not differ significantly over the study period (Table 2). UPDRS I, II and IV scores did not change significantly between both groups over the study period, nor did the total UPDRS scores (Table 2). Means and 95% confidence intervals of differences compared to baseline for each visit are plotted in Fig 2.

**Table 2:** Main results in the placebo / bee venom groups.

	Placebo	Bee venom	P-value
**Primary criterion**			
UPDRS III at V13: variation after injection			
- Median, Interquartile range	1 ; 4.5	1 ; 2	0.86
- Mean, standard deviation	0.35 ; 2.85	0.72 ; 2.37	
Change over time from V2 to V13 for criteria monthly evaluated*			
UPDRS III before injection	- 0.11 (0.08)	-0.03 (0.08)	0.23
UPDRS I before injection	-0.03 (0.02)	-0.015 (0.018)	0.67
UPDRS II before injection	-0.087 (0.04)	0.013 (0.04)	0.08
UPDRS IV before injection	0.009 (0.02)	- 0.023 (0.02)	0.32
Hoehn and Yahr stages	-0.0009 (0.0001)	-0.0007 (0.0001)	0.3
Schwab and England scores	0.005 (0.003)	-0.001 (0.003)	0.09
**Variation between V14 and V1 for criteria evaluated at the inclusion and at the end of study**			
BREF			
- Median, interquartile range	0 ; 1	0 ; 0.5	0.22
- Mean, standard deviation	0 ; 1.5	-0.2 ; 1	
MMS			
- Median, interquartile range	0 ; 2	0 ; 1	0.13
- Mean, standard deviation	- 0.2 ; 1.6	0.7 ; 1.04	
LED			
- Median, interquartile range	51 ; 129	0 ; 162.7	0.22
- Mean, standard deviation	98; 156	64 ; 127	
PDQ-39			
- Median, interquartile range	-1.5 ; 9.2	2.2; 13.5	0.6
- Mean, standard deviation	-1.2 ; 5.4	-0.3; 7.5	

* Data are presented with estimates of monthly variation and its standard error (regression parameters of the linear mixed model for repeated data)

### Secondary outcomes

Both BREF (batterie rapide d'évaluation frontale) and MMS scores improved in the bee venom compared to the placebo group between V2 and V13, albeit non-significantly (Table 2). No differences were seen in change over time of Schwab and England scores and Hoehn and Yahr stages (Table 2).

When comparing total PDQ-39 scores (Table 2), no significant differences could be noted between the bee venom and placebo group over the study period. However, in the ADL (activities of daily living) subscale, patients in the bee venom group fared worse than those in the placebo group (p = 0.05).

The assessment of tremor and rigidity using a segmental rating scale [17] showed no difference of evolution over the study period between the treatment groups in temporal or localization scores. (Table 2). The maximal amplitude of rest tremor (p < 0.05) as well as action and postural tremor (p < 0.02), however, decreased in the placebo group compared to controls.

### Dopamine transporter imaging

Mean BP values in all regions of interest decreased between V1 and V13 in both groups consistent with a progression of dopaminergic denervation along time. However, changes were not significantly different between the two groups (Table 3).

**Table 3:** Comparison of [123I]-FP-CIT binding potential changes between V2 and V13 in the placebo / bee venom groups.

Region	Placebo (mean ± standard deviation)	Bee venom (mean ± standard deviation)	P-value
Caudate (right hemisphere)	-0.111 ± 0.194	-0.093 ± 0.193	0.56
Caudate (left hemisphere)	-0.121 ± 0.210	-0.079 ± 0.188	0.41
Anterior putamen (right hemisphere)	-0.115 ± 0.215	-0.108 ± 0.206	0.97
Anterior putamen (left hemisphere)	-0.062 ± 0.138	-0.037 ± 0.198	0.62
Posterior putamen (right hemisphere)	-0.019 ± 0.163	-0.067 ± 0.170	0.62
Posterior putamen (left hemisphere)	-0.026 ± 0.202	-0.010 ± 0.194	0.99

### Kinetics of bee venom specific IgE and IgG4

**Fig 3:**
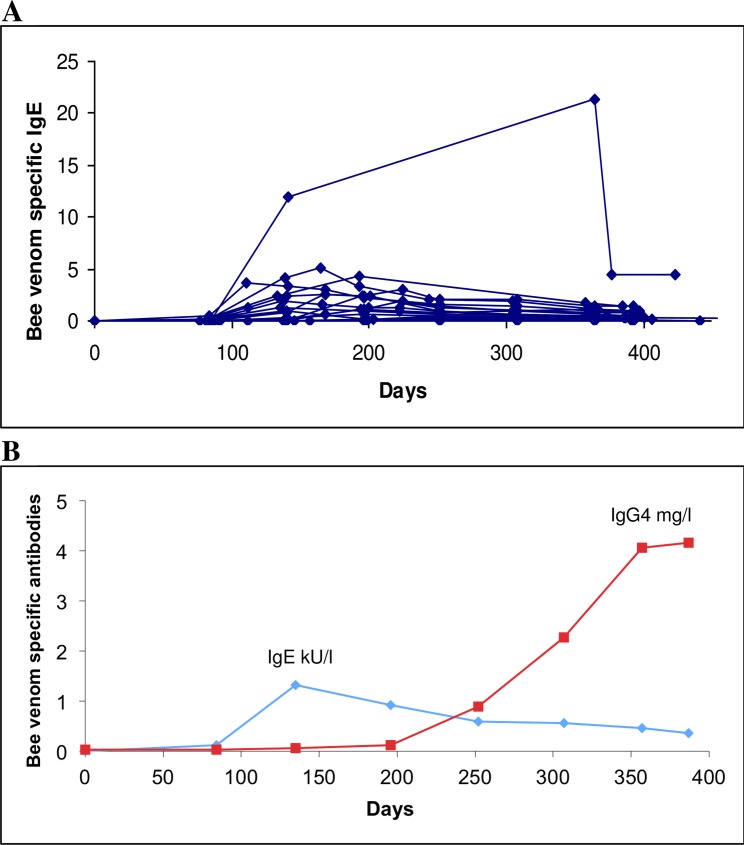
Kinetics of bee venom specific antibodies. The sample at day -60 was taken at the pre-screening, the first bee venom injection was done at day 0 and the first sample after at day 30. Diamonds are for specific IgE (kU/L) and the squares for specific IgG4 (mg/L). (A)kinetics of specific IgE production for all patients; (B) kinetics for specific IgE and IgG4 for a single representative patient.

The 20 control patients and two patients out of 20 who received bee venom injections did not produce either specific IgE or IgG4. The remaining 18 patients produced specific IgE, four patients after the first bee venom injection (20%), five after the second (25%), and nine after the third injection (45%). The peak of antibody synthesis was reached after the fifth injection for half of the patients and then antibody concentration started to decrease (Fig 3A). Bee venom specific IgG4 were detected in 12 patients out of 20 (60%). However, specific IgG4 were not determined in all samples of each patient. The kinetics of antibody production were available for six patients: three (50%) produced antibodies before the sixth injection and the remainder (50%) thereafter. The production of IgG4 antibodies was delayed an average of three months to that of IgE antibodies, and was correlated with a decrease of the latter (Fig 3B).

### Safety/tolerance / adverse effects

We classified four adverse events as severe, namely positive ST in patients treated with bee venom and which led to their exclusion from the trial, one at V6, one at V7 and two at V8. However, it must be noted that this was purely a precautionary measure as we never observed anything corresponding to an allergic reaction. Other than that, we recorded the following light adverse effects: redness/itching at the injection site (placebo: 6 / bee venom: 165), insomnia (placebo: 1 / bee venom: 1), nausea (placebo: 9 / bee venom: 3), fatigue (placebo: 10 / bee venom: 2), dyskinesia (placebo: 1 / bee venom: 1), bradycardia (placebo: 2 / bee venom: 0).

## Discussion

After an 11 month period of monthly administration, a single injection of bee venom did not significantly decrease UPDRS III scores in the « off » condition. Also, UPDRS III scores over the course of 11 months, and nuclear imaging, did not evolve significantly differently between both treatment groups. LED increased less in the bee venom group compared to placebo but again, differences between both groups failed to reach significance. Finally, motor fluctuations and dyskinesia remained unaffected by bee venom treatment over the study period.

One other clinical study has investigated the potential symptomatic effect of bee venom on PD symptoms [13]. Using bee venom acupuncture twice a week for eight weeks at 10 acupuncture points (compared to an acupuncture group without bee venom and a control group), a significant decrease of UPDRS III scores from 15.0 to 10.0 points was observed (17.0 to 13.0 in the acupuncture group, n = 13, p<0.05; 13.0 to 13.0 in the control group, n = 9). However, this study is hardly comparable to ours as it remains unclear to what extent acupuncture and bee venom contributed respectively to this effect. Also, the quantities of bee venom used were not quantified in micrograms (0.01 mL bee venom diluted to 0.005% at each injection point) and it thus remains unclear how much product was actually injected.

Cognition as assessed by the MMS appeared to be slightly enhanced under bee venom but not significantly compared to placebo. Although the MMS is a rough tool to investigate cognitive function, it is interesting to note that hippocampal SK channel blockade has been suggested to enhance cognitive performance [20–22]. Recently, in 6-OHDA-lesioned rats, apamin i.p. has been shown to display pro-cognitive effects, in addition to reversing motor deficits and attenuating anxiety-related behavior [7].

Surprisingly, the ADL subscores on the PDQ-39 scale improved significantly over the study period in the placebo group compared to the bee venom group. We can only speculate about the reasons for these changes given that they do not correlate with motor scores. Most likely, the local inflammatory reactions frequently observed after bee venom injections may contribute to this effect. As such, they were generally not debilitating but nonetheless anxiogenic since patients usually wished to know whether these reactions were due to bee venom or not, a question we were of course unable to answer during the study period due to blinding.

The decrease of amplitude of rest, positional and action tremor using the segmental rating scale in the placebo group was equally unexpected. At baseline, patients in the placebo group had more severe postural and kinetic tremor than those who were to be treated with bee venom. We suppose that the difference at the end of the study between the groups is partly due to a floor effect among patients who received bee venom and had milder tremor. In addition to more severe positional and kinetic tremor, at baseline control patients had higher Hoehn and Yahr stages, higher UPDRS III ratings, higher LED, lower scores on the Schwab and England scale, and a longer duration of manifest PD. We therefore think that patients in the control group were slightly more affected. Possibly as a consequence, LED showed a trend towards higher increase over the study period among control patients.

Regarding safety, despite the production of specific IgE antibodies in response to bee venom injection, no clinical symptoms of allergy were detected. The protocol we used seems safe and was consistent with the highest dose recommended by the manufacturer for immunotherapy, 100 μg in a single injection. About 45% of patients produced IgE before the third injection and the remainders after. The peak of IgE synthesis was observed after the fifth injection in half of patients and IgG4 production began subsequently to be detected in half of patients after the sixth injection. Thus, early in the immune response a critical phase may exist which may cause allergy with clinical symptoms. However, over time, a balance in the level of specific IgE and IgG4 antibodies is induced. This phenomenon explains the desensitization of allergic patients treated by immunotherapy and may also put to rest fears of using bee venom in non-allergic subjects, which is a important finding as such and paves the way for future studies.

This study has several limitations. First, because of safety concerns, we stuck to a typical desensitization protocol for allergies against bee venom. To achieve more potent effects of bee venom, it is likely necessary to increase the dosage and/or to decrease the injection interval, for instance to at least fortnightly or even weekly injections. In traditional apitherapy with live bees (personal communications), patients are treated weekly or bi-weekly with up to four stings per session which makes for a comparative dose 16 times higher than the one used in our study. Also, in a randomized cross-over trial of bee stings in multiple sclerosis, patients were stung by up to 20 bees three times per week, pushing the comparative dose to 90 times that used in our study [23]; note that in this study, no allergic reactions occurred in the 25 patients who completed the trial over a 6 month period. However, no routine IgE monitoring or STs were performed. Finally, in our own MPTP mouse study, bee venom was administered twice weekly with dosages of 120 μg/kg body weight, thereby ranging up to 100 times higher than in humans [6]. Second, based on the observed results, the study was underpowered. When we planned the study, we overestimated the difference between groups. The observed size effect was 0.14 while we planned the study with a size effect equal to one. Regarding drop outs, it must be underlined that these patients were excluded based on very and possibly overly strict safety concerns (see above) as no allergic reactions actually occurred. However, to avoid any preventable risk, we decided to be extremely conservative in our exclusion policy. Third and lastly, patients in the bee venom group were less severely affected than the patients in the placebo group, which might have distorted/diminished possible treatment effects.

As bee venom is a potentially lethal substance in case of an anaphylactic reaction, two options appear feasible to design a safer substance: first, to use apamin as a monotherapy. However, apamin is currently not licensed for any medical condition and would therefore have to undergo phase I toxicology testing before a phase II trial could be considered. Of note, animal data suggest that the therapeutic range of apamin may be narrower as a single substance than when embedded in whole bee venom. In MPTP-treated mice, high dose apamin treatment induced motor symptoms akin to tremor or dyskinesia [6]. In SK3-deficient mice, symptoms reminiscent of psychosis could be observed [24]. Also, apamin seemed to induce less neuroprotective effects than whole bee venom in our mouse study, suggesting that other components of bee venom might contribute to this effect [6]. One candidate, for instance, is mellitin which has been shown to slow motor neuron degeneration in a murine amyotrophic lateral sclerosis model [25]. Therefore, an alternative strategy might be to remove the allergens from bee venom. The most potent allergens in bee venom is phospolipase A_2_, followed by hyaluronidase and icarapin [26]. Also, weaker allergens such as mellitin or ingredients whose allergenicity remain unknown (vitellogenin) are present in bee venom [26]. Thus, allergen removal is probably a challenging process. Moreover, it cannot be excluded that these also contribute to potential symptomatic and/or neuroprotective effects of bee venom [27].

In summary, our study did not evidence any clear symptomatic or disease-modifying effects of monthly bee venom injections over an 11 month period compared to placebo using a standard bee venom allergy desensitization protocol in PD patients. Of note, bee venom administration appeared safe in non-allergic patients. Given the increasingly strong preclinical rationale that SK channel blockade may be beneficial in treating PD short and long term, we believe that a larger study with less stringent exclusion criteria regarding IgE levels and ST is warranted. In particular, we feel that higher administration frequency and possibly higher individual doses of bee venom may reveal its potency in treating PD.

## Supporting Information

S1 Dataset Dataset. (7Z)

S1 Fig CONSORT checklist. (DOC)

S1 Protocol Protocol. (DOC)

## Abbreviations

ADL: activities of daily living
BP: binding potential
BREF: batterie rapide d'évaluation frontale
DBS: deep brain stimulation
GPi: globus pallidus internus
LED: levodopa-equivalent daily dose
MMS: Mini Mental State
MNI: Montreal Neurological Institute
MPTP: 1-methyl-4-phenyl-1,2,3,6-tetrahydropyridine
PD: Parkinson disease
PDQ: Parkinson's Disease Questionnaire
SK channels: Ca^2+^-activated small conductance K^+^ channels
SNpc: substantia nigra pars compacta
SNpr: substantia nigra pars reticulata
ST: skin test
STN: subthalamic nucleus
UPDRS: United Parkinson’s Disease Rating Scale
VOI: volumes of interest
