# Correction: Bee Venom for the Treatment of Parkinson Disease – A Randomized Controlled Clinical Trial

**DOI:** 10.1371/journal.pone.0162937

**Published:** 2016-09-08

**Authors:** Andreas Hartmann, Julia Muellner, Niklaus Meier, Helke Hesekamp, Priscilla van Meerbeeck, Marie-Odile Habert, Aurélie Kas, Marie-Laure Tanguy, Merry Mazmanian, Hervé Oya, Nissen Abuaf, Hafida Gaouar, Sabrina Salhi, Fanny Charbonnier-Beaupel, Marie-Hélène Fievet, Damien Galanaud, Sophie Arguillere, Emmanuel Roze, Bertrand Degos, David Grabli, Lucette Lacomblez, Cécile Hubsch, Marie Vidailhet, Anne-Marie Bonnet, Jean-Christophe Corvol, Michael Schüpbach

The second author's name is spelled incorrectly. The correct name is: Julia Muellner. The correct citation is: Hartmann A, Muellner J, Meier N, Hesekamp H, van Meerbeeck P, Habert M-O, et al. (2016) Bee Venom for the Treatment of Parkinson Disease – A Randomized Controlled Clinical Trial. PLoS ONE 11(7): e0158235. doi:10.1371/journal.pone.0158235
