# Supplementary material for: Bee Venom for the Treatment of Parkinson Disease – A Randomized Controlled Clinical Trial
Source: PLoS One. 2016 Jul 12;11(7):e0158235. doi: 10.1371/journal.pone.0158235 (PMC4942057; doi:10.1371/journal.pone.0158235)

**
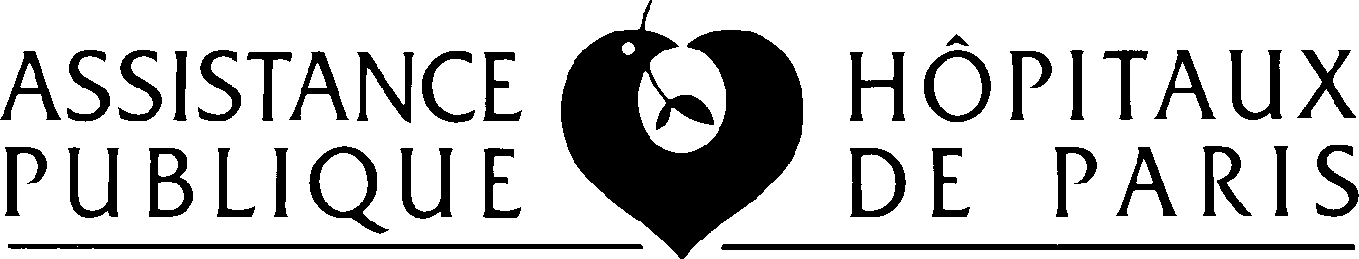
**

Evaluation des effets SYMPTOMATIQUES et neuroprotecteurs du venin d’abeille dans le traitement de la maladie de Parkinson : ETUDE **MIREILLE**

Essai randomisé en double aveugle versus placebo

Version n°2.0 du 05/10/2010

| **Promoteur : Assistance Publique – Hôpitaux de Paris (AP-HP)**  Direction de la Politique Médicale  Service de la Recherche Clinique et du Développement  (Délégation Régionale de la Recherche Clinique)  Carré Historique de l’Hôpital Saint-Louis  1, Avenue Claude Vellefaux – 75010 Paris  www.drrc.ap-hop-paris.fr |
| --- |
| **Investigateur coordonnateur: Dr Andreas HARTMANN**  Centre d’Investigation Clinique – Pitié-Salpêtrière  Hôpital de la Pitié-Salpêtrière  47 Bd de l’Hôpital  75651 Paris Cedex 13  Tél. : 01 42 16 19 50/ Fax : 01 42 16 19 58  Email : andreas.hartmann@psl.aphp.fr |
| **Responsable scientifique :**  **Dr Michael SCHÜPBACH**  Centre d’Investigation Clinique – Pitié-Salpêtrière  Hôpital de la Pitié-Salpêtrière  47 Bd de l’Hôpital  75651 Paris Cedex 13  Tél. : 01 42 16 19 50/ Fax : 01 42 16 19 58  Email : michael.schupbach@wanadoo.fr |
| **Chef de projet DRCD : Yannick VACHER**  DRCD - Hôpital Saint Louis  1, Avenue Claude Vellefaux - 75010 Paris  Tél. : 01 44 84 17 30/ Fax : 01 44 84 17 99  Email : yannick.vacher@sls.aphp.fr |
| **URC en charge de l’étude** : Responsable : **Professeur Alain MALLET**  Hôpital de la Pitié-Salpêtrière  47 Bd de l’Hôpital  75651 Paris Cedex 13  Tel : 01 42 16 05 88/ Fax : 01 42 16 05 13  E-mail : [alain.mallet@psl.aphp.fr](mailto:philippe.lechat@psl.aphp.fr) |
|  |

**Investigateurs :**

**Centre d’Investigation Clinique – Pitié-Salpêtrière**

Dr Andreas HARTMANN/ andreas.hartmann@psl.aphp.fr

Dr. Michael SCHÜPBACH/ michael.schupbach@wanadoo.fr

Dr Anne-Marie BONNET/ anne-marie.bonnet@psl.aphp.fr

Dr Jean-Christophe CORVOL/ jean-christophe.corvol@psl.aphp.fr

**Equipes associées** :

Réalisation des Imageries TEMP

Dr Marie-Odile HABERT

Service de Médecine Nucléaire (Pr. André AURENGO)

Hôpital de la Pitié-Salpêtrière

47 Bd de l’Hôpital

75651 Paris Cedex 13

Tel : 01 42 17 62 81/ Fax : 01 42 17 62 92

Email : [marie-odile.habert@psl.aphp.fr](mailto:marie-odile.habert@psl.aphp.fr)

**Experts**

Pr. Francisque LEYNADIER

Dr. Thierry HADDAD

Dr. Hafida GAOUAR
Centre d'Allergie, Hôpital Tenon
4 rue de la Chine
75970 Paris Cedex 20

Tel : 01 56 01 72 29/ Fax : 01 56 01 64 58

Email : [francisque.leynadier@tnn.aphp.fr](mailto:francisque.leynadier@tnn.aphp.fr), [thierryhaddad@free.fr](mailto:thierryhaddad@free.fr), [hafida.gaouar@tnn.aphp.fr](mailto:hafida.gaouar@tnn.aphp.fr)

**URC en charge de l’étude** :

Responsable : Professeur Alain MALLET

Hôpital de la Pitié-Salpêtrière

47 Bd de l’Hôpital

75651 Paris Cedex 13

Tel : 01 42 16 05 88/ Fax : 01 42 16 05 13

E-mail : [alain.mallet@psl.aphp.fr](mailto:philippe.lechat@psl.aphp.fr)

Coordination de l’étude : Dr Merry MAZMANIAN

Tel: 01 42 16 84 31/ Fax: 01 42 16 24 40/ [merry.mazmanian@psl.aphp.fr](mailto:merry.mazmanian@psl.aphp.fr)

ARC de l’étude : Dr Hervé OYA

Tél : 01 42 16 24 39/ Fax : 01 42 16 24 40/ [herve.oya@psl.aphp.fr](mailto:herve.oya@psl.aphp.fr)

**RESUME DU PROTOCOLE**

**Titre :** Evaluation des effets symptomatiques et neuroprotecteurs du venin d’abeille dans le traitement de la maladie de Parkinson : essai randomisé en double aveugle versus placebo

**Objectifs de la recherche :**

- Quantifier la magnitude d’un potentiel effet symptomatique à long terme suite à l’injection de venin d’abeille, évaluée grâce à l’évolution du score moteur de l’échelle UPDRS (UPDRS III) avant et après injection, à la visite M11.

- Étudier l’influence d’une thérapie par venin d’abeille (100 μg/mois) sur la progression de la maladie de Parkinson (score moteur de l’échelle UPDRS (UPDRS III).

- Corréler la progression des symptômes avec la dénervation nigrostriatale observée par imagerie SPECT (DaTSCAN).

- Quantifier l’évolution (apparition, progression ou régression) de fluctuations motrices sur la durée de l’étude suite au traitement pas venin d’abeille (score de fluctuations motrices de l’échelle UPDRS (UPDRS IV).

**Critères d’inclusion:**

- - patient ayant une maladie de Parkinson idiopathique d’après les critères de la Parkinson’s Disease Society Brain Bank (Hughes *et al.*, 1992)
  - âge > 40 ans (exclusion de formes juvéniles)
  - stade Hoehn and Yahr 1,5-3 en « off »
  - DaTSCAN pathologique
  - IRM excluant des formes secondaires ou atypiques de syndromes parkinsoniens
  - négatif à l’intradermoréaction pour le venin d’abeille
  - affiliation à un régime de Sécurité Sociale (bénéficiaire ou ayant droit)
  - capable de comprendre et de signer le consentement éclairé

**Critères de non-inclusion :**

- - maladie de Parkinson au stade Hoehn & Yahr < 1,5 ou > 3
  - Intradermoréaction positive contre le venin d’abeille
  - IgE spécifiques positifs au venin d’abeille
  - Allergie connue au venin d’abeille
  - contre-indications au traitement par venin d’abeille Alyostal
  - syndrome parkinsonien atypique (vérifié par IRM)
  - traitement en cours ou dans les 6 derniers mois par neuroleptique sauf dompéridone
  - insuffisance rénale ou hépatique ou anomalie significative sur le bilan sanguin ou l’ECG effectué lors de l’étude
  - SPECT normal
  - contre-indications à la réalisation d’un examen SPECT (grossesse) + IRM (port d’un objet métallique sur ou dans le corps et claustrophobie)
  - lésion cérébrale d’origine vasculaire sévère, tumorale ou infectieuse (vérifié par IRM)
  - patiente enceinte ou allaitante et femmes en âges de procréer sans contraception efficace
  - patient présentant un syndrome dépressif majeur ou souffrant d'une affection psychiatrique (critères du DSM IV) non-traités
  - troubles cognitifs responsables d’un syndrome démentiel ou mettant en cause l’information éclairée du patient et la signature du consentement
  - patient bénéficiant d’une mesure de protection légale
  - défaut de signature du formulaire de consentement

**Nombre de sujets nécessaire :** 44 patients

**Durée de participation de chaque sujet :** 14 mois maximum

**Durée de la période d’inclusion :** 18 mois

**Durée totale de la recherche :** 32 mois

**Durée de la période d’exclusion**: un mois après la dernière injection du produit.

**Méthodologie :**

Étude monocentrique, contrôlée, en double aveugle, en groupes parallèles, comparant l’effet d’une thérapie par venin d’abeille contre placebo chez les patients parkinsoniens avec traitement symptomatique concomitant au stade Hoehn et Yahr 1,5-3, après 11 mois de traitement.

**Critère d’évaluation principal :**

Quantifier la magnitude d’un **potentiel effet symptomatique** à long terme suite à l’injection de venin d’abeille, évaluée grâce à l’évolution du score moteur de l’échelle UPDRS (UPDRS III) avant et après injection à M11.

**Critères d’évaluations secondaires :**

- Étudier l’influence d’une thérapie par venin d’abeille (100 μg/mois) sur la **progression de la maladie de Parkinson**. Cette progression sera évaluée grâce à l’évolution du score moteur de l’échelle UPDRS (UPDRS III). Une mesure annexe sera l’évolution des doses concomitantes du traitement dopaminergique.
- **Corréler la progression des symptômes avec la dénervation nigrostriatale observée par imagerie SPECT (DaTSCAN).** La dénervation du striatum sera examinée avec une gammacaméra 3 têtes en utilisant un ligand DAT (Amersham™) qui estime la perte de synapses dopaminergiques. Ces données d’imagerie seront corrélées avec les données cliniques obtenues à l’aide de l’UPDRS III.
- Quantifier l’évolution (apparition, progression ou régression) de **fluctuations motrices** sur la durée de l’étude suite au traitement pas venin d’abeille. Cette évolution sera évaluée grâce à l’évolution du score de fluctuations motrices de l’échelle UPDRS (UPDRS IV).

## 2–Page de signature du protocole

**Page de SIGNATURE D'UN PROTOCOLE de recherche**

**par l’investigateur coordonnateur et le représentant du promoteur**

*Recherche Biomédicale N° DRRC :* code :P090102

Titre : Evaluation des effets symptomatiques et neuroprotecteurs du venin d'abeille dans le traitement de la maladie de Parkinson : essai randomisé en double aveugle versus placebo

Version N° 2.0 du : 05/10/2010

| **L’investigateur coordonnateur :** |  |
| --- | --- |
| HARTMANN, Andreas, Dr | Date : ……………/………/……….. |
| Centre d´Investigation Clinique  Fédération des Maladies du Système Nerveux  Hôpital de la Pitié-Salpêtrière  47 Boulevard de l´Hôpital  75651 Paris Cedex 13 | Signature : |
| Le responsable scientifique : |  |
| SCHÜPBACH, Michael, Dr | Date : ……………/………/……….. |
| Centre d´Investigation Clinique  Fédération des Maladies du Système Nerveux  Hôpital de la Pitié-Salpêtrière  47 Boulevard de l´Hôpital  75651 Paris Cedex 13 | Signature |
| **Le promoteur :** |  |
| Christophe MISSE |  |
| Assistance Publique – Hôpitaux de Paris  Délégation régionale à la recherche clinique | Date : ……………/………/……….. |
| Hôpital Saint Louis | Signature : |
| 75010 PARIS |  |

**3- Introduction et justification de la recherche**

#### *Un besoin en molécules neuroprotectrices dans la maladie de Parkinson*

Malgré l’existence d’un grand nombre de traitements symptomatiques efficaces pour la maladie de Parkinson (MP), il n’existe à ce jour aucun traitement permettant de freiner, voire d’arrêter la progression de la perte neuronale dopaminergique dans la substance noire qui détermine cette pathologie.

Nous avons identifié un patient parkinsonien avancé (15 ans de maladie, stade Hoehn et Yahr 3-4) qui est apiculteur. Suite à une réaction allergique au venin d’abeille début 2004, ce patient subit une injection mensuelle de **venin d’abeille**. Suite à chaque injection, le score moteur (United Parkinson’s Disease Rating Scale (UPDRS) III) du patient s’améliore de 70% en quelques heures et lui permet subséquemment de supprimer entièrement son traitement par L-dopa durant deux semaines. Dans les deux semaines jusqu’à la prochaine injection, son traitement par L-Dopa a diminué de 50% par rapport à la période précédant sa cure de désensibilisation. Ceci suggère que le venin d’abeille possède une activité aussi bien **symptomatique** que **neurorestauratrice** dans la maladie de Parkinson.

Cette observation clinique peut trouver sont explication dans des données récentes concernant la physiopathologie de la MP. Ainsi, les neurones dopaminergiques de la substance noire se caractérisent par des propriétés électriques singulières leur permettant d’optimiser la libération de dopamine dans striatum. Dans la MP, il a été proposé que les neurones dopaminergiques souffraient d’un déficit d’excitabilité les rendant progressivement silencieux jusqu’à ce qu’ils meurent. Cette activité est contrôlée par des canaux potassiques qui réduisent l’activité électrique des neurones dopaminergiques nigraux. En conséquent, le blocage des ces canaux augmentent l’activité électrique des ces neurones. Un polypeptide particulièrement efficace dans ce blocage est **l’apamine**, un des composants principaux du venin d’abeille. C’est donc par le biais de l’action de l’apamine que pourrait s’expliquer l’effet du venin d’abeille sur la régression des symptômes dans la MP.

Concernant l’utilisation du venin d’abeille ou de l’apamine dans la MP, une **demande de brevet** a été déposée auprès de l’INPI (Demande de Brevet d’Invention en France N° 07 04754, le 2 juillet 2007 par l’AP-HP : Médicament pour traiter la maladie de Parkinson. Auteurs: Andreas Hartmann, Anne-Marie Bonnet, Michael Schüpbach).

Nous souhaitons conduire une **étude clinique de Phase II** afin de valider ou falsifier cette observation clinique singulière. En cas de résultats positifs de cette étude, l’invention serait substantiellement valorisée. Nous proposons donc de conduire une étude randomisée, contrôlé par placebo et en double aveugle en traitant deux groupes de patients parkinsoniens (n=20/groupe, stade Hoehn & Yahr 1,5-3) comprenant une visite mensuelle sur 12 mois pour injection de venin d’abeille (100 μg/injection) et une évaluation clinique. Les « end-points » cliniques seront multiples : (i) scores moteurs (UPDRS) ; (ii) échelles de dyskinésies ; (iii) qualité de vie ; (iv) doses équivalentes de L-Dopa consommées à M0 et M12 ; (v) une étude en SPECT DaTSCAN afin de disposer d’une mesure objective de la dénervation dopaminergique striatale.

## 4- Données de la littérature et pré-requis

*Physiopathologie de la maladie de Parkinson*

Les signes moteurs classiques de la MP (akinésie, rigidité et tremblement de repos) sont la conséquence d’une dégénérescence lente et progressive des neurones dopaminergiques de la substance noire, un noyau situé dans le mésencéphale. Les données accumulées au cours des 20 dernières années indiquent que la mort de ces neurones est un événement multifactoriel. Parmi les cibles thérapeutiques potentielles identifiées, les plus solides à ce jour sont (i) un déficit du complexe I de la chaîne respiratoire mitochondriale (Schapira, 2008) et (ii) le stress oxydatif (Gandhi et Wood, 2005). Le dernier découlant (en partie) du premier, une des conséquences fonctionnelles de la libération de radicaux libres d’origine mitochondriale pour les neurones dopaminergiques sont un efflux de potassium de l’espace intra- vers l’espace extracellulaire : l’activité électrique du neurone s’en trouve réduite (vide infra).

Les neurones dopaminergiques du mésencéphale possèdent des propriétés membranaires intrinsèques qui leur permettent de décharger de manière spontanée *in vivo* en modes différents: pacemaker, aléatoire (“random”) et bursts (Fig. 1).


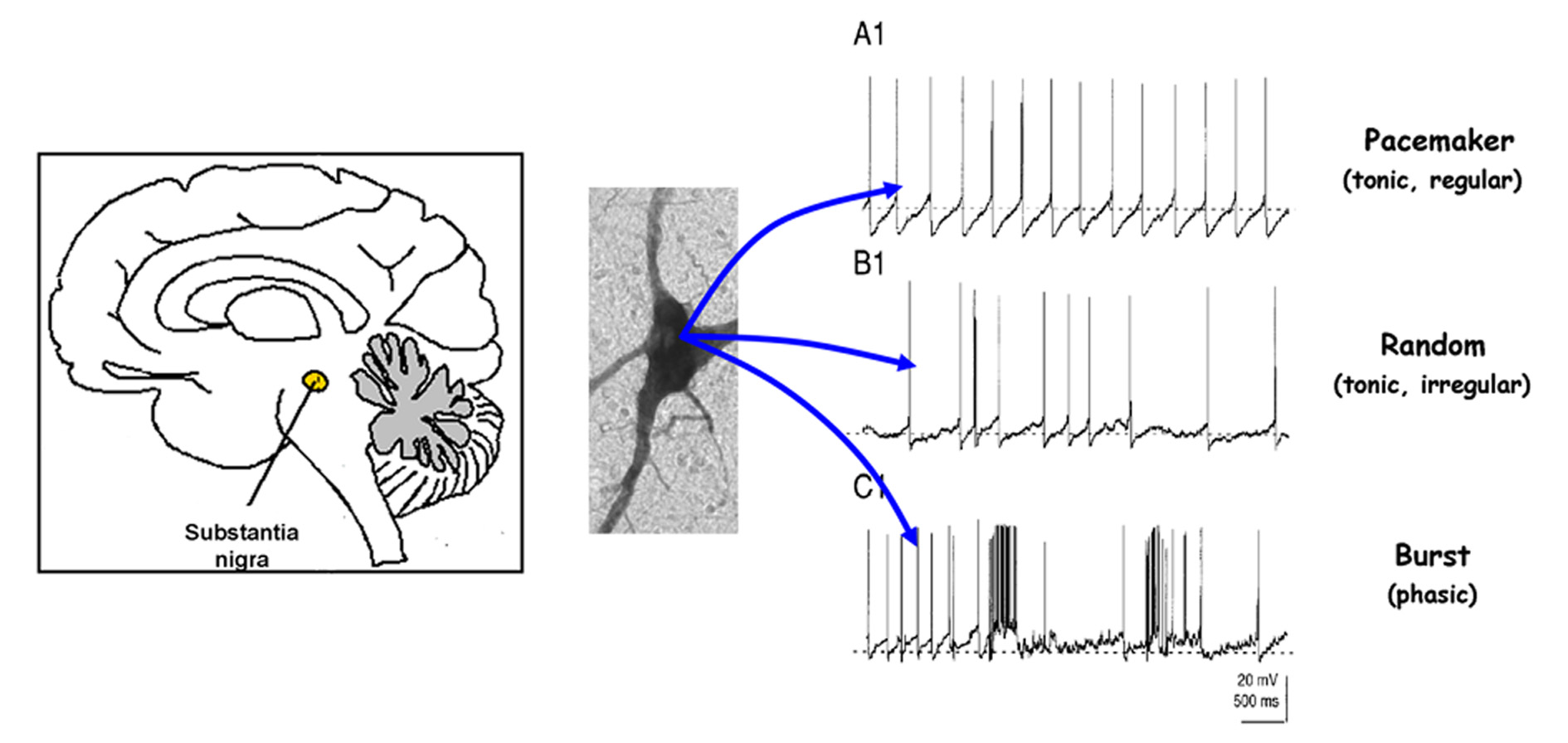
**Figure 1**

Ces modes de décharge sont contrôlés par des interactions complexes de canaux ioniques ou à récepteurs (Wolfart et Roeper, 2002) et permettent aux neurones dopaminergiques d’optimiser le relargage de dopamine dans la zone cible, le striatum (Gonon et Buda, 1985). Par ailleurs, plusieurs études suggèrent que l’activité des neurones dopaminergiques est non seulement cruciale pour le relargage de dopamine mais aussi pour leur survie, démonstration réalisée *in vitro* sur cultures de mésencéphale post-mitotiques (Salthun-Lassalle *et al.*, 2004, 2005). Ces observations ont suscité l’hypothèse que l’altération des courants ioniques pourraient, dans certaines conditions, participer à la mort des neurones dopaminergiques du mésencéphale.

Spécifiquement, il a été proposé que dans la MP, les neurones dopaminergiques souffraient d’un déficit d’excitabilité qui les rend progressivement silencieux jusqu’à leur mort. Cette hypothèse a été étudiée à l’aide de canaux potassiques adénosine triphosphate-sensitifs K (K_ATP_) (Ashcroft et Gribble, 1998). L’activation continue des ces canaux K_ATP_ induit la mort des neurones dopaminergiques du mésencéphale; inversement, la délétion de la sous-unité Kir6.2 de ces canaux dans la souris protège les neurones dopaminergiques et leurs axones contre l’action du 1-méthyl-4-phényl-1,2,3,6-tétrahydropyridine (MPTP), une toxine spécifique des neurones dopaminergiques (Liss *et al.*, 2005). Des enregistrements ‘patch-clamp’ dans des tranches de mésencéphale de souris contrôles ont pu montré que l’inhibition du complexe I de le chaîne respiratoire mitochondriale par le 1-méthyl-4-phénylpyridinium (MPP^+^), le métabolite actif du MPTP, était à l’origine de l’activation des canaux K_ATP_ channels dans les neurones dopaminergiques de la substance noire, ce qui rendait ceux-ci progressivement hyperpolarisés et silencieux. De plus, l’hyperpolarisation des neurones dopaminergiques médiées par les canaux K_ATP_ et induites par l’inhibition du complexe I pouvait être abolie par le capteur de radicaux libres MnTBAP, ce qui indique que des radicaux libres d’origine mitochondriale peuvent activer les canaux K_ATP_ dans des neurones dopaminergiques (Michel *et al.*, 2006, 2007)(Fig. 2).


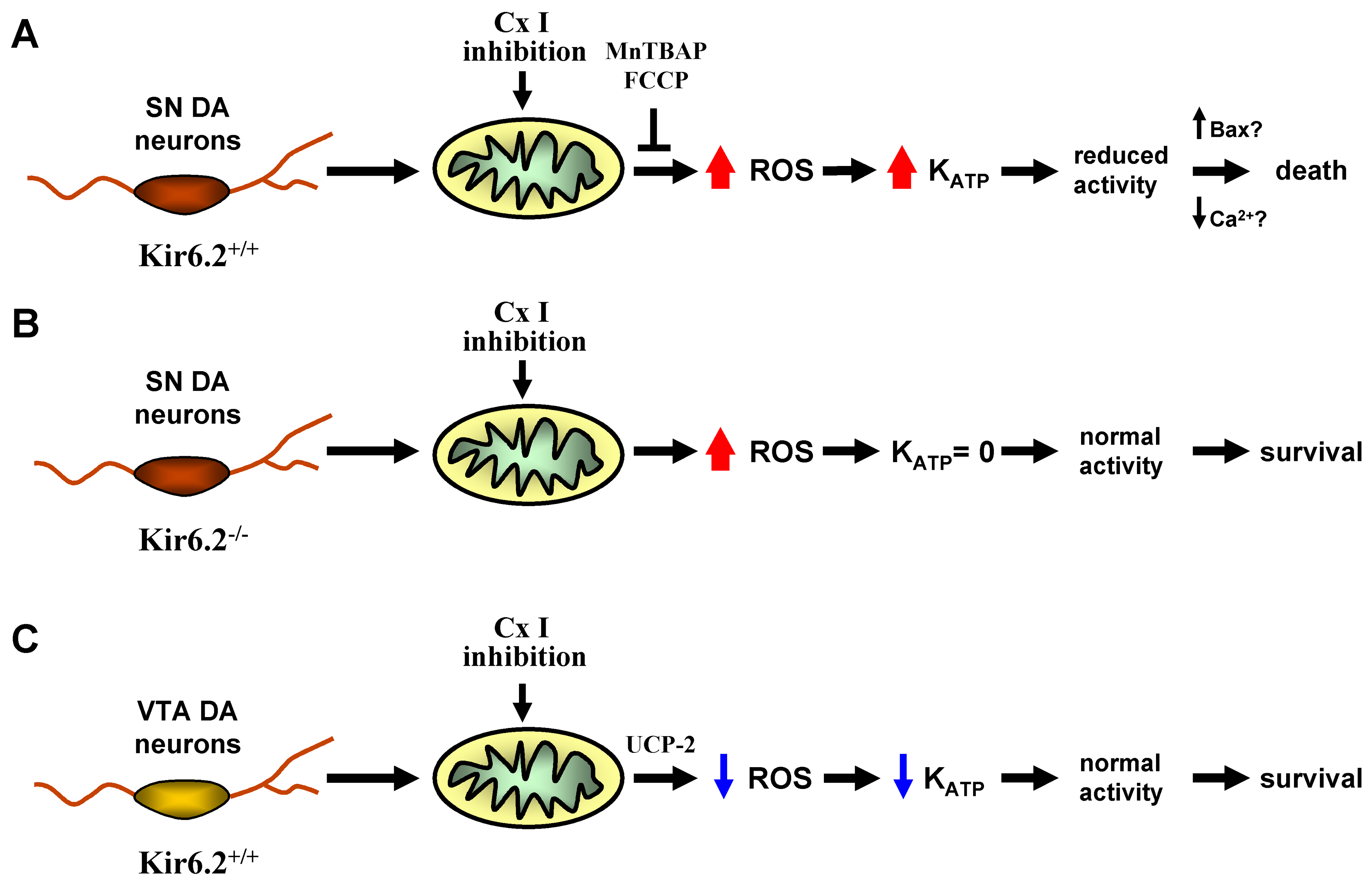


**Figure 2**

Une autre manière d’empêcher l’hyperpolarisation des neurones dopaminergiques de la substance noire est de bloquer l’activité d’un autre type de canaux potassiques que les canaux K_ATP_: ces canaux s’appellent canaux SK et sont activés par le calcium. Ces canaux [de sous-type SK3 dans les neurones dopaminergiques de la substance noire] possèdent un rôle majeur dans le contrôle du switch entre un mode de décharge tonique et en burst en condition physiologique (Waroux *et al.*, 2005). Ainsi, le blocage de canaux SK par l’apamine, une toxine présente dans le venin d’abeille, diminue la dégénérescence spontanée de neurones dopaminergiques dans un modèle de cultures primaires mésencéphaliques dissociées (Salthun-Lassalle *et al.*, 2004). En revanche, il n’existe pas de données à ce jour concernant l’effet de l’apamine sur la survie des neurones dopaminergiques de la voie nigrostriatale *in vivo*. Néanmoins, il est possible de spéculer qu’une augmentation de l’activité électrique des neurones dopaminergiques s’effectue par une synthèse accrue de la tyrosine hydroxylase (El Mestikawy *et al.*, 1985), l’enzyme de synthèse limitant de la dopamine. Ce mécanisme ne concernerait pas seulement les neurones dopaminergiques encore “sains” dans la MP (mécanisme *neuroprotecteur*), mais aussi les neurones dits “souffrants”, c’est-à-dire en voie de dégénérescence (mécanisme *neurorestaurateur*). Il est estimé que 10-15% des neurones dopaminergiques dans la substance noire des patients parkinsoniens autopsiés font partie de cette population (Hirsch *et al.*, 1988). En théorie, donc, la réactivation métabolique de ces neurones par augmentation de l’activité électrique semble possible.

*Pré-requis et résultats préliminaires*

Ces résultats obtenus dans des modèles expérimentaux de la MP confortent une observation clinique récente. Un patient parkinsonien suivit dans notre service depuis 15 ans (MP avancée, stade Hoehn & Yahr 3-4 en 2001 et programmé à cette époque pour une stimulation bilatérale à haute fréquence su noyau sous-thalamique) montre une amélioration nette et rapide de ses symptômes moteurs suite à des injections mensuelles de venin d’abeille dans le cadre d’un protocole de désensibilisation (initié en 2004 à cause d’une allergie au venin d’abeille). Cet effet apparaît dans les 30 minutes suivants l’injection sous-cutanée d’une dose de 100 μg de venin d’abeille (dose standard dans les protocoles de désensibilisation) avec une baisse concomitante des score UPDRS III de 70%. Après ces injections mensuelles, le patient ne nécessite plus de traitement dopaminergique pendant au moins deux semaines. Dans les deux semaines suivantes, jusqu’à la prochaine injection de venin d’abeille, ses doses de L-Dopa augmentent progressivement mais n’atteignent au final que 50% des doses nécessaires avant la cure de désensibilisation: ceci suggère non seulement une effet neuroprotecteur du venin d’abeille, mais un effet *neurorestaurateur*. Par ailleurs, ce patient n’a plus de fluctuations motrices ni d’instabilité posturale et montre une régression globale de son score Hoehn & Yahr vers le stade 2.

Basé sur les données expérimentales présentées ci-dessus, *nous supposons que ces effets bénéfiques du venin d’abeille sont médiés par l’apamine*. Il est important de noter que l’apamine est la seule neurotoxine polypeptidergique capable de pénétrer la barrière hémato-encéphalique après injection systémique (18 acides aminés; séquence: Cys-Asn-Cys-Lys-Ala-Pro-Glu-Thr-Ala-Leu-Cys-Ala-Arg-Arg-Cys-Gln-Gln-His-NH_2_; formule moléculaire: C_79_H_131_N_31_O_24_S_4_, poids moléculaire: 2027 Da).

#### *Imagerie fonctionnelle dans la maladie de Parkinson*

Plusieurs études récentes de neuroprotection dans la maladie de Parkinson ont utilisé l’imagerie par ^123^β-CIT SPECT (single photon émission computed tomography) afin de déterminer la progression de la perte de l’innervation dopaminergique nigrostriatale (cf. Parkinson Study Group 2000), ceci afin de disposer d’un marqueur « objectif » de la dégénérescence de cette voie. En effet, les mesures cliniques (UPDRS) reflètent aussi, en particulier en début de maladie, des phénomènes compensatoires et sous-estiment ainsi la perte neuronale. Concernant les ligands disponibles pour visualiser le système dopaminergique nigrostriatal, les ligands du transporteur membranaire de la dopamine (DAT) estiment directement la densité des synapses dopaminergiques striatales, et reflètent de manière précise et sensible le degré de dénervation nigrostriatale chez les patients parkinsoniens (Ribeiro *et al.*, 2002). Un ligand DAT (^123^I^-^FP-CIT, vide infra) pour utilisation avec des caméras SPECT est commercialisé par Amersham™, le DaTSCAN. Il possède l’avantage d’une relative homogénéité par rapport à d’autres ligands de fabrication « on site » (Tolosa *et al.*, 2007).

## 5- Objectifs de la recherche (hypothèses testées, objectif primaire, objectifs

## secondaires)

### Hypothèses testées

La thérapie par venin d’abeille possède un effet symptomatique prolongé et ralentit la progression de la maladie de Parkinson.

### Objectif primaire

Montrer que l’injection sous-cutanée mensuelle de 100 µg de venin d’abeille a un **effet symptomatique** dans la maladie de Parkinson, évaluée grâce à l’évolution du score moteur de l’échelle UPDRS (UPDRS III) avant et après injection par rapport au placebo à M11

**c. Objectifs secondaires**

- Etudier l’effet symptomatique du venin d’abeille à chaque temps (chaque mois) pour savoir si l’effet est constant au cours du temps, s’il existe un épuisement ou au contraire une potentialisation de l’effet symptomatique.
- **Étudier l’influence d’une** thérapie par venin d’abeille (100 µg/mois) sur la **progression de la maladie de Parkinson**. Cette progression sera évaluée grâce à l’évolution du score moteur de l’échelle UPDRS (UPDRS III) en off. Une mesure annexe sera l’évolution des doses concomitantes du traitement dopaminergique.
- **Corréler la progression des symptômes avec la dénervation nigrostriatale observée par imagerie SPECT (DaTSCAN).** La dénervation du striatum sera examinée avec une gammacaméra 3 têtes en utilisant un ligand DAT (Amersham™) qui estime la perte de synapses dopaminergiques. Ces données d’imagerie seront corrélées avec les données cliniques obtenues à l’aide de l’UPDRS III et l’échelle motrice segmentale.
- Quantifier l’évolution (apparition, progression ou régression) des complications **fluctuations motrices** sur la durée de l’étude suite au traitement pas venin d’abeille. Cette évolution sera évaluée grâce à l’évolution du score de complications fluctuations motrices de l’échelle UPDRS (UPDRS IV).

## 6- Plan expérimental

### a. Choix du plan expérimental

Étude monocentrique, contrôlée, en double aveugle, en groupes parallèles, comparant l’effet de l’injection sous-cutanée mensuelle de 100 µg de venin d’abeille pendant 11 mois versus placebo chez des patients parkinsoniens avec traitement symptomatique concomitant au stade Hoehn et Yahr 1,5-3, après 11 mois de traitement.

*Évaluation clinique*

- Les patients seront recrutés sur une période de 18 mois dans notre consultation de neurologie (Fédération des Maladies du Système Nerveux, Pitié-Salpêtrière). En se basant sur notre expérience, nous nous attendons à pouvoir recruter entre 40 et 50 patients parkinsoniens au stade Hoehn et Yahr 1,5-3. Ces patients seront évalués mensuellement en consultation, de M-2 à M12, avec une visite de pré-screening (M-2) et de sélection (M-1) avant la première injection de venin d’abeille (M0). Lors de la visite de pré-screening, les taux d'IgE spécifiques au venin d’abeille seront déterminés pour détecter une éventuelle allergie préexistante au venin. Un deuxième contrôle sera effectué après la première injection de venin d’abeille ainsi que tous les deux mois après le début de l’étude. Pour cela, les patients devront être sevrés de leurs traitements anti-parkinsoniens. Concrètement, ils devront prendre leur dernière prise de levodopa la soirée précédent la visite. Si un traitement par agoniste dopaminergique est en place, la période de sevrage devra être de cinq demi-vies minimum. En cas de besoin, l’agoniste dopaminergique pourra être remplacé par des doses équivalentes de levodopa.

Ces consultations se tiendront au Centre d´Investigation Clinique (INSERM CIC 9503) de la Pitié-Salpêtrière. À chaque visite (hormis à V0), un bilan biologique, un ECG et l’examen des signes vitaux seront effectués, ainsi qu’un interrogatoire sur les effets indésirables éventuels. Les échelles standard appliquées seront l’UPDRS I-IV, l’échelle de Hoehn & Yahr, de Schwab & England et l’échelle segmentale. Aussi, nous emploierons, plus ponctuellement, les échelles de MMS, BREF et PDQ-39.

*L’imagerie par SPECT*

Les examens SPECT seront réalisés au Service de Médecine Nucléaire, Hôpital de la Pitié-Salpêtrière. Les patients seront examinés à M-1 et à M12. **Le ligand** sera administré en I.V. avec une dose moyenne injectée de l’ordre de 111 à 185 Mbq. Les images SPECT seront acquises sur une durée de 30 minutes 3 heures après injection du ligand, sur une gammacaméra 3 têtes (IRIX, Philips). Aucun prélèvement sanguin n’est nécessaire au cours de l’examen.

Les images SPECT seront recalées sur l’IRM des patients acquise en 3D T1 sur une machine 3T (Siemens). Cette IRM sera faite dans le cadre de la recherche au Centre de neuroimagerie de recherche (CENIR – www.cenir.org) à l’Hôpital de la Pitié-Salpêtrière sauf si elle est déjà réalisé au cours des trois derniers mois. L’analyse d’images sera faite selon deux approches différentes: 1) une analyse voxel-par-voxel en utilisant le logiciel SPM2 (statistical parametric mapping, <http://www.fil.ion.ucl.ac.uk/spm/spm2.html>). Cette analyse sera restreinte par un masque aux changements survenus dans le striatum et le mésencéphale (« small volume correction »). Cette analyse déterminera l’évolution de la perte dopaminergique pendant la durée de l’étude dans les deux bras (placebo/traitement neuroprotecteur) et déterminera les différences d’évolution entre ces groupes par une ANOVA (décrit dans Whone *et al.*, 2003 et Whone *et al*., 2004). 2) Une approche par “régions d’intérêt” utilisant le programme Anatomist® qui permettra de subdiviser le striatum en sous-régions sphériques de 5-6 mm de diamètre et de déterminer l’uptake de ligand par sous-région et de le corréler à la progression de la maladie. Dans les deux cas, la variable étudiée sera le potentiel de liaison : (As/Ans) – 1 où As est l’activité spécifique mesurée dans le striatum, et Ans l’activité non spécifique mesurée dans le cortex occipital.

Cette analyse permettra d’obtenir trois mesures principales:

- Le **mapping** **striatal** du potentiel de liaison du ^123^I-FP-CIT reflètera la perte de terminaisons dopaminergiques striatales.
- La **progression** de la perte de signal au cours d’une année chez chaque individu ainsi que la moyenne du groupe. Ceci nous permettra d’identifier des sous-régions du striatum dans lesquelles les changements majeurs s’effectuent au cours de l’évolution et au début de la maladie.
- La **corrélation** entre l’évolution segmentale des symptômes et le changement de fixation du ^123^I-FP-CIT dans des sous-régions du striatum. Cette analyse nous permettra de mieux apprécier les changements cliniques subtils qui sont associés à une dénervation dopaminergique nigrostriatale spécifique.

### b. Critères de sélection des personnes

##### Critères d’inclusion

- - patient ayant une maladie de Parkinson idiopathique d’après les critères de la Parkinson’s Disease Society Brain Bank (Hughes *et al.*, 1992)
  - âge > 40 ans (exclusion de formes juvéniles)
  - stade Hoehn and Yahr 1,5-3 en « off »
  - DaTSCAN pathologique
  - IRM excluant des formes secondaires ou atypiques de syndromes parkinsoniens
  - négatif à l’intradermoréaction pour le venin d’abeille (sans dépasser la concentration de 1 µg/ml avec un cut off idéal à 0,1 µg/ml)
  - affiliation à un régime de Sécurité Sociale (bénéficiaire ou ayant droit)
  - capable de comprendre et de signer le consentement éclairé

##### Critères de non-inclusion :

- - maladie de Parkinson au stade Hoehn & Yahr < 1,5 ou > 3
  - Intradermoréaction positive contre le venin d’abeille (sans dépasser la concentration de 1 µg/ml avec un cut off idéal à 0,1 µg/ml)
  - taux des IgE spécifiques positifs
  - allergie connue au venin d’abeille
  - contre-indications au traitement par venin d’abeille (Alyostal®) : réactions inhabituelles (rénales, musculaires, articulaires) après piqûre d’hyménoptères ; déficits immunitaires sévères Cachexie, affections malignes, néphropathies, vascularite systémique (dont périartérite noueuse), déficience immunitaire grave, phase aiguë d'un état morbide quelconque. Traitement par immunosuppresseurs ou corticoïdes, traitement par les bêtabloquants.
  - syndrome parkinsonien atypique (atrophie multisystématisée, paralysie supra-nucléaire progressive, autre syndrome extrapyramidal) (vérifié par IRM)
  - traitement en cours ou dans les 6 derniers mois par neuroleptique sauf dompéridone
  - insuffisance rénale ou hépatique ou anomalie significative sur le bilan sanguin ou l’ECG effectué lors de l’étude
  - SPECT normal
  - contre-indications à la réalisation d’un examen SPECT (grossesse) + IRM (port d’un objet métallique sur ou dans le corps et claustrophobie)
  - lésion cérébrale d’origine vasculaire sévère, tumorale ou infectieuse
  - patiente enceinte ou allaitante et femmes en âges de procréer sans contraception efficace
  - patient présentant un syndrome dépressif majeur ou souffrant d'une affection psychiatrique (critères du DSM IV) non traités
  - troubles cognitifs responsables d’un syndrome démentiel ou mettant en cause l’information éclairée du patient et la signature du consentement
  - patient bénéficiant d’une mesure de protection légale.
  - défaut de signature du formulaire de consentement

### c. Mode de recrutement

Le recrutement sera assuré à la consultation de la Fédération des Maladies du Système Nerveux à l’Hôpital de la Pitié-Salpêtrière par les médecins neurologues investigateurs ou par les neurologues leur signalant les patients acceptant de participer à cette étude et répondant aux critères d’inclusion. Nous estimons à plus de 1000 le nombre de patients parkinsoniens suivis de façon régulière aux consultations citées par les médecins neurologues investigateurs par an. Le recrutement de 44 patients (40 + 4, voir infra) sur 18 mois correspond donc environ à 3% de ces consultations.

### Nombre prévu de personnes et justification

Le nombre de patients à inclure est de 40 patients. Le nombre de patients à sélectionner est évalué à 44 patients (+10% par rapport à 40) car il faut prévoir des patients sélectionnés (ayant signé le consentement) qui ne seront pas randomisés s’ils ne répondent pas à tous les critères.

La justification de ce nombre de patients est détaillée plus bas (chapitre 11c).

### Durée de participation de chaque personne ayant accepté de participer à la recherche et durée d’exclusion

Délai entre visite de pré-screening et la visite de 1^ère^ injection du patient = 2 mois maximum.

La durée totale de l’étude pour chaque patient est donc de 14 mois maximum.

## 7- Schéma et conduite de la recherche :

**Inclusion**

Randomisation (réalisé par l’investigateur entre VI et V2)

**↓** Première injection

**↓**

V0 V1 V2 V3 V4 V5 V6 V7 V8 V9 V10 V11 V12 V13 V14

├───┴──┴───┴───┴───┴───┴───┴───┴───┴───┴───┴───┴───┴──┘

↑

↑ **Sélection**

**Pré-screening**

### a. Suivi des patients

La chronologie et le contenu des visites sont résumés en annexe 1. Un intervalle de +/- 3 jours est acceptable entre la date théorique de la visite de suivi du patient et la date réelle de la visite.

##### Chronologie des visites

- V0 : visite de pré-screening (IDR et IgE spécifique au venin d’abeille)
- V1 : visite de sélection et SPECT
- V2 : visite de première injection (M0)
- V3 – V13 : visites de suivi
- V14 (12 mois après randomisation = M12) : visite de fin d’étude et SPECT

##### Contenu des visites

1- Visite de pré-screening (V0)

- signature du consentement éclairé
- vérification des critères d’inclusion et de non-inclusion (en particulier l’intradermoréaction réalisée par le médecin investigateur qui aura été préalablement formé par le service d’Allergologie de l’Hôpital Tenon)
- prise de sang pour la détermination du taux d'IgE spécifiques au venin d’abeille

2 – Visite de sélection (V1) – si IDR au venin d’abeille et IgE spécifiques au venin d’abeille négatifs

- examen clinique (poids, taille), signes vitaux
- prise de sang pour bilan biologique (hémogramme, plaquettes, transaminases, ionogramme, créatininémie)
- Mini Mental State (MMS) (cf. annexe 6)
- Batterie rapide d'évaluation frontale (BREF) (cf. annexe 10)
- UPDRS I – IV (cf. annexe 2)
- Echelle ségmentale (cf. annexe 8)
- Echelle Schwab & England (cf. annexe 7)
- Stade Hoehn & Yahr (cf. annexe 3)
- ECG
- IRM 3T au CENIR (si résultat non disponible)
- Transport au Service de Médecine Nucléaire, Hôpital de la Pitié-Salpêtrière pour SPECT le jour même, si possible (maximum une semaine après la visite d’inclusion ; réalisation du SPECT au ^123^I-FP-CIT). Une carte patient (cf. annexe 5) sera remise au patient à cette visite.

Une fois les critères de sélection validés, le patient est contacté pour lui confirmer sa participation à l’étude. Un rendez vous lui sera fixé pour recevoir sa première injection. L’investigateur aura au préalable randomisé son patient afin que la pharmacie prépare les traitements.

3 – Visite de première injection (V2)

- Consultation selon le rendez vous donné ; le patient n’a pas pris de traitement symptomatique le matin
- ECG, signes vitaux
- Prise de sang : hémogramme, plaquettes, transaminases, ionogramme, créatininémie
- Parkinson's Disease Questionnaire (PDQ-39 ; qualité de vie) (cf. annexe 9)
- UPDRS I – IV (partie III avec vidéo). Cette vidéo sera analysée en aveugle après la fin de participation du patient à l’étude.
- Echelle ségmentale
- Echelle Schwab & England
- Stade Hoehn & Yahr
- Injection sous-cutanée (100 μg venin d’abeille ou placebo ; divisés en deux injections de 50 μg à intervalle d’une heure)
- Observation du patient pendant au moins deux heures après les deux injections de venin d’abeille/placebo
- ECG et signes vitaux après deux heures.
- UPDRS III (avec vidéo)

4 – Visites de suivi (V3 – V13)

- Consultation selon le rendez vous donné ; le patient n’a pas pris de traitement symptomatique le matin
- ECG, signes vitaux
- Prise de sang : hémogramme, plaquettes, transaminases, ionogramme, créatininémie
- Vérification de la dose du traitement anti-parkinsonien pris depuis la dernière visite
- Interrogatoire spécifique sur la tolérance du traitement
- UPDRS I – IV (V13 partie III avec vidéo pré et post-injection venin d’abeille)
- Echelle ségmentale
- Echelle Schwab & England
- Stade Hoehn & Yahr
- Injection sous-cutanée (100 μg venin d’abeille ou placebo)
- Observation du patient pendant au moins deux heures
- ECG, signes vitaux
- UPDRS III (avec vidéo). Cette vidéo sera analysée en aveugle après la fin de participation du patient à l’étude.

5 – Visite de fin d’étude (V14)

- Consultation selon le rendez vous donné ; le patient n’a pas pris de traitement symptomatique le matin
- ECG, signes vitaux
- Prise de sang : hémogramme, plaquettes, transaminases, ionogramme, créatininémie, détermination du taux d'IgE spécifiques au venin d’abeille
- Vérification de la dose du traitement anti-parkinsonien pris depuis la dernière visite
- Interrogatoire spécifique sur la tolérance du traitement
- Mini Mental State (MMS)
- Batterie rapide d'évaluation frontale (BREF)
- Parkinson's Disease Questionnaire (PDQ-39 ; qualité de vie)
- UPDRS I – IV
- Echelle ségmentale
- Echelle Schwab & England
- Stade Hoehn & Yahr
- Transport au Service de Médecine Nucléaire, Hôpital de la Pitié-Salpêtrière : réalisation de l’examen SPECT au ^123^I-FP-CIT

##### Actes, examens et prélèvements

Toutes les prises de sang seront réalisées au Centre d’Investigation Clinique de l’Hôpital de la Pitié-Salpêtrière.

- Une prise de sang pour bilan biologique à V1 et V14 (NFS, plaquettes, ionogramme, bilan hépatique) et pour les taux d’IgE spécifiques soit 4 tubes de 6 ml (24 ml de sang prélevé pour les visites V0 (juste IgE spécifique), V1, V3, V5, V7, V9, V11 et V14).
- Concernant les visites V2 à V13, un bilan biologique (NFS, plaquettes, ionogramme, bilan hépatique) sera réalisé à chaque fois, soit 3 tubes de 6 ml (18 ml de sang prélevé pour les visites V2 à V13).
- Au total environ 270 ml seront prélevés dans le cadre de cette étude.

##### Lieux de réalisation des examens, des prélèvements et des dosages

Toutes les visites (V0 – V14) seront réalisées au Centre d’Investigation Clinique (CIC) de l’Hôpital de la Pitié-Salpêtrière.

Les prélèvements sanguins seront effectués par les infirmières du CIC.

Les dosages de biologie standard seront effectués dans les laboratoires d’hématologie (NFS, plaquettes) et de biochimie (ionogramme, bilan hépatique) du GHPS. Les tubes seront acheminés aux laboratoires par pneumatique. Les dosages des IgE spécifiques seront réalisés dans le laboratoire de Biochimie de l’Hôpital Tenon.

### b. Randomisation des patients

Après vérification des critères d’inclusion et de non-inclusion, les patients éligibles seront randomisés avant la visite V2.

La randomisation sera centralisée et réalisée par voie électronique (randoWeb). Chaque investigateur pourra accéder au site de randomisation au moyen d’un mot de passe personnel.

Les investigateurs seront en aveugle du groupe de traitement.

La randomisation sera gérée par le Pôle Biostatistique, Méthodologie et Gestion des données de l’Unité de Recherche Clinique, Groupe Hospitalier Pitié-Salpêtrière-Charles-Foix. Un fax doublé d’un mail de randomisation seront envoyés à la pharmacie de l’hôpital via RandoWeb.

### c. Durée totale prévisionnelle de la recherche

La durée totale de l’étude pour chaque patient est de 14 mois maximum.

La période de recrutement prévue est de 18 mois à raison de 4 à 6 patients inclus par mois.

La durée totale prévisionnelle de l’étude est de 32 mois.

### d. Fin d’étude

Les patients finissent l’étude après la visite de fin d’étude à M12.

### e. Sortie prématurée d’étude

Tout patient présentant au moins un des items suivants sortira de l’étude :

- Demande de sortie d’étude exprimée par le patient

- Situation d’urgence nécessitant l’interruption de l’étude selon l’investigateur en particulier si

l’augmentation du taux d’IgE spécifique dépasse un certain seuil

## 8- Médicaments

Le médicament utilisé dans le cadre de cette étude (**ALYOSTAL®** 110 µg venin d'abeille) est hors AMM pour la maladie de Parkinson, mais possède une AMM pour la désensibilisation à l’allergie au venin d’abeille. Le flacon d’Alyostal® 110 µg permet de délivrer après reconstitution 100 µg de venin d’abeille dans 1 ml.

Les données sur le médicament (VIDAL 2009) sont détaillées dans l’annexe 4.

La pharmacie recevra un fax et un mail via RandoWeb précisant le numéro de randomisation du patient lui même défini par ses initiales, sa date de naissance et son numéro d’inclusion. La pharmacie recevra en début d’étude la liste de randomisation. La pharmacie préparera pour chaque visite le coffret de traitement (actif ou placebo) portant le numéro d’identification du patient. Ce coffret sera délivré au vue de l’ordonnance à l’interne en pharmacie du CIC ou à l’infirmière en ouvert (hors protocole) du CIC. Chaque seringue sera préparée au CIC par l’interne en pharmacie du CIC ou par une infirmière en ouvert du CIC selon le protocole de reconstitution du Vidal.. La première administration nécessitera la préparation de deux seringues de 50 μg à injecter par voie sous cutanée à intervalle de une heure par un des investigateurs en aveugle du bras de traitement.

Le placebo sera administré de la même manière et il sera constitué de NaCl 0.9%.

Préservation de l’aveugle

Des coffrets patients seront préparés par la pharmacie dès la randomisation des patients. Elle délivrera à l’interne pharmacie du CIC ou à l’infirmière en ouvert au CIC les coffrets patient, au vue de l’ordonnance délivrée par l’investigateur. L’interne en pharmacie du CIC ou l’infirmière en ouvert préparera les seringues de traitement et mettra une étiquette portant le numéro d’identification patient sur chaque seringue. Les seringues seront distribuées à l’infirmière en aveugle du CIC pour administration.

Intra dermo réaction (IDR)

Pour les IDR, un flacon d’Alyostal 110 μg sera attribué par patient ainsi que l’histamine et les flacons de solvant phénolé à 0,4 %. La pharmacie mettra en dotation des flacons d’Alyostal, d’histamine et de solvant phénolé au CIC qui complétera alors une fiche de traçabilité par patient.

## 9- Critères d’évaluation

### a. Variables mesurées

##### i. Variable mesurée pour l’objectif primaire :

- Différence du score UPDRS III avant et après injection de venin d’abeille à la visite M11

##### ii. Variables mesurées pour les objectifs secondaires :

- Différence du score UPDRS III obtenu à M0 et à M12
- SPECT
- Evolution du score UPDRS IV de M0 à M12

### b. Méthodologie pour l’étude clinique

UPDRS III, détaillés ci-dessus dans le annexe 2.

### c. Méthodologie pour l’étude d’imagerie SPECT

L’analyse d’images permettra d’obtenir des images paramétriques du potentiel de liaison du ^123^I-FP-CIT en utilisant une modélisation dite de Logan, déjà validée pour ce traceur (Poyot *et al.*, 2001). Ces images paramétriques seront utilisées directement pour l’analyse voxel par voxel (SPM) ou pour le calcul des valeurs dans les régions d’intérêt. La valeur obtenue permet d’estimer le nombre de transporteurs disponibles dans chaque voxel (ou région). C’est cette valeur régionale ou voxel par voxel qui est comparée au sein de chaque groupe (progression) et entre les groupes (différence des progressions) par une ANOVA bi-directionnelle.

## 10- Gestion des évènements indésirables graves

## a- Description des paramètres d’évaluation de la sécurité :

*i- Evènement indésirable*

Toute manifestation nocive survenant chez une personne qui se prête à une recherche biomédicale que cette manifestation soit liée ou non à la recherche ou au produit sur lequel porte cette recherche.

*ii- Effet indésirable d’un médicament expérimental*

Toute réaction nocive et non désirée à un médicament expérimental quelle que soit la dose administrée

*iii- Evènement ou effet indésirable grave*

Tout évènement ou effet indésirable qui entraîne la mort, met en danger la vie de la personne qui se prête à la recherche, nécessite une hospitalisation ou la prolongation de l’hospitalisation, provoque une incapacité ou un handicap importants ou durables, ou bien se traduit par une anomalie ou une malformation congénitale, et s’agissant du médicament, quelle que soit la dose administrée.

*iv- Effet indésirable inattendu d’un médicament expérimental*

Tout effet indésirable dont la nature, la sévérité ou l’évolution ne concorde pas avec les informations figurant dans le résumé des caractéristiques du produit lorsque le médicament est autorisé, et dans la brochure pour l’investigateur lorsqu’il n’est pas autorisé.

*v- Fait nouveau*

Toute nouvelle donnée de sécurité, pouvant conduire à une réévaluation du rapport des bénéfices et des risques de la recherche ou du médicament expérimental, ou qui pourrait être suffisant pour envisager des modifications dans l’administration du médicament expérimental, dans la conduite de la recherche.

Tout fait nouveau survenu dans la recherche ou dans le contexte de la recherche, provenant des données de la littérature ou des recherches en cours devra être notifié au promoteur.

**b- Procédures mises en place en vie de l’enregistrement et de la notification des**

**évènements indésirables**

### *i- Evènements indésirables non-graves*

Les évènements indésirables, recueillis par l’investigateur lors de l’interrogatoire médical doivent être reportés dans le cahier d’observation à la section prévue à cet effet.

Un seul évènement doit être reporté par item. Seront précisés les circonstances de survenue, le délai d’apparition par rapport aux prises de médicament, leur évolution et leur prise en charge médicale ainsi que les explorations effectuées pour les caractériser.

Les effets indésirables de survenue éventuellement tardive seront recueillis lors de la visite de clôture d’étude avec notamment les échelles UPDRS II (activités de la vie quotidienne) et IV (complications du traitement), et le PDQ-39 (qualité de vie).

Les évènements indésirables non graves seront recueillis lors des visites V2 – V14. En cas de survenue d’évènements indésirables entre deux visites, les patients peuvent contacter à tout instant les infirmières du CIC (01 42 16 18 16) ou le médecin de garde du CIC (06 74 35 17 96).

*ii- Evènements indésirables graves (EIG)*

Les investigateurs doivent notifier immédiatement au promoteur AP-HP les évènements indésirables graves tels que définis ci-dessus.

L'investigateur complète les formulaires d’évènements indésirables graves (du cahier d'observation de la recherche) et les envoie au DRCD par fax au **01 44 84 17 99** et ce, dans les **48 heures** (après si possible un appel téléphonique immédiat au 01 44 84 17 23 en cas de décès ou de menace vitale)

L’investigateur doit également informer l’URC en charge de la recherche de la survenue de l’EIG.

Pour chaque évènement indésirable grave, **l’investigateur devra émettre un avis sur le lien de causalité de l’évènement avec chaque médicament expérimental et les autres traitements éventuels.**

L’obtention d’informations relatives à la description et l’évaluation d’un évènement indésirable peuvent ne pas être possibles dans le temps imparti pour la déclaration initiale.

**Aussi, l'évolution clinique ainsi que les résultats des éventuels bilans cliniques et des examens diagnostiques et/ou de laboratoire, ou toute autre information permettant une analyse adéquate du lien de causalité seront rapportés :**

- **soit sur la déclaration initiale d’EIG s’ils sont immédiatement disponibles,**
- **soit ultérieurement et le plus rapidement possible, en envoyant par fax une nouvelle déclaration d’EIG complétée (et en précisant qu’il s’agit d’un suivi d’EIG déclaré et le numéro de suivi).**

Toutes les déclarations faites par les investigateurs devront identifier chaque sujet participant à la recherche **par un numéro de code unique** attribué à chacun d’entre eux.

**En cas de décès notifié** d’un sujet participant à la recherche**, l’investigateur communiquera au promoteur tous les renseignements complémentaires demandés** (compte-rendu d’hospitalisation, résultats d’autopsie…).

Tout fait nouveau survenu dans la recherche ou dans le contexte de la recherche, provenant de données de la littérature ou de recherches en cours, devra être notifié au promoteur

#### - Déclaration des évènements indésirables graves aux Autorités de Santé

#### Elle sera assurée par le Pôle de Pharmacovigilance du DRCD, après évaluation de la gravité de l’évènement indésirable, du lien de causalité avec chaque médicament expérimental et les autres traitements éventuels ainsi que du caractère inattendu des effets indésirables.

Toutes les suspicions d’effet indésirable grave inattendu seront déclarées par le promoteur aux autorités compétentes dans les délais légaux.

**Toute donnée de sécurité ou tout fait nouveau qui pourrait modifier significativement l’évaluation du rapport des bénéfices et des risques d’un médicament expérimental, ou de la recherche, ou qui pourrait conduire à envisager des modifications concernant l’administration du médicament ou la conduite de la recherche, sera transmise par le promoteur aux autorités compétentes, au Comité de Protection des Personnes et aux investigateurs de la recherche.**

### c. Levée de l’aveugle

En cas de nécessité, l’investigateur pourra demander à tout moment une levée de l’aveugle pour un patient à la pharmacie de la Pitié Salpêtrière qui est en ouvert et possède la liste de randomisation. La pharmacie pourra donc réaliser la levée d’insu en cas d’urgence sur la base du N° de randomisation attribué au patient 24h/24h.

**11- Gestion des données et statistiques**

Tout patient randomisé et ayant reçu au moins une injection de venin d’abeille sera inclus dans les analyses. Toutes les analyses seront réalisées en intention de traiter.

**a. Analyse principale**

Le critère principal est la variation du score UPDRS III entre avant et après injection de venin d’abeille à la visite V13 (M11). Cette évaluation sera faite par un médecin en aveugle du bras de traitement et qui n’a pas participé à l’inclusion et au suivi du patient en exploitant la vidéo de l’UPDRS III à (M11).

Les deux groupes seront comparés par le test non paramétrique de Mann-Whitney.

En cas d’arrêt de traitement en cours d’étude, le patient sera considéré en échec et la variation du score attribuée à ces patients sera nulle.

**b. Analyses secondaires**

- Les cinétiques des variations du score UPDRS III avant et après injection de venin d’abeille pendant la durée de traitement (V2 à V13) seront comparées à l’aide d’un modèle de régression mixte avec effet sujet aléatoire.

- Les variations sur 12 mois (V2 à V14) des scores à l’UPDRS III et à l’UPDRS IV seront comparées au moyen d’un modèle de régression mixte avec effet sujet aléatoire.

Concernant les analyses secondaires et pour les patients qui arrêteront leur traitement, les données prises en compte seront les données recueillies jusqu’à l’arrêt.

**c. Nombre prévu de personnes à inclure dans la recherche**

Les hypothèses ayant servi de base au calcul du nombre de sujets nécessaires sont les suivantes :

- Le score moyen de l’UPDRS III à V13 avant l’injection est estimé à 28 points.

- La différence de variation avant et après l’injection entre les deux groupes est supposée égale à 20% (différence de 5,6 points).

- Le coefficient de variation est supposé égal à 1 (soit un écart-type de la variation du score de 5,6 points supposé identique dans les deux groupes).

Le test de comparaison prévu étant le test non paramétrique de Mann-Whitney, les hypothèses ci-dessus conduisent à une estimation de la probabilité qu’un patient du groupe placebo présente une augmentation du score plus basse qu’un patient du groupe traité de 0,240. Sous ces hypothèses et pour un risque de première espèce de 5%, l’inclusion de 20 patients dans chaque groupe est nécessaire pour assurer à l’étude une puissance de 80%.

Le nombre de patients à sélectionner est évalué à 44 patients (+10% par rapport à 40) car il faut prévoir des patients sélectionnés (ayant signé le consentement) qui ne seront pas randomisés s’ils ne répondent pas à tous les critères.

**d. Risque de première espèce**

Tous les tests seront bilatéraux et conduits au seuil de 5%.

**e. Critères statistiques d'arrêt de la recherche**

Aucun critère statistique d’arrêt n’est défini.

**f. Gestion des données**

La gestion des données de l’étude sera sous la responsabilité de l’Unité de Recherche Clinique Pitié-Salpêtrière/Charles Foix. Les données recueillies dans les cahiers d’observation feront l’objet d’une double saisie indépendante. Le fichier de données sera déclaré à la CNIL dans le cadre d’une déclaration normale.

**12- Droit d’accès aux données et documents source**

Les personnes ayant un accès direct conformément aux dispositions législatives et réglementaires en vigueur, notamment les articles L.1121-3 et R.5121-13 du code de la santé publique (par exemple, les investigateurs, les personnes chargées du contrôle de qualité, les moniteurs, les assistants de recherche clinique, les auditeurs et toutes personnes appelées à collaborer aux essais) prennent toutes les précautions nécessaires en vue d'assurer la confidentialité des informations relatives aux médicaments expérimentaux, aux essais, aux personnes qui s'y prêtent et notamment en ce qui concerne leur identité ainsi qu’aux résultats obtenus. Les données collectées par ces personnes au cours des contrôles de qualité ou des audits sont alors rendues anonymes.

**13- Contrôle et assurance de la qualité**

**La recherche sera encadrée selon les procédures opératoires standard du promoteur.**

Le déroulement de la recherche dans les centres investigateurs et la prise en charge des sujets seront faits conformément à la déclaration d’Helsinki et les Bonnes Pratiques Cliniques en vigueur.

**a. Procédures de monitoring**

Il s’agit d’une recherche de risque C, le pourcentage, de dossiers à monitorer est de 100%

Les ARC représentants du promoteur effectueront des visites des centres investigateurs au rythme correspondant au schéma de suivi des patients dans le protocole, aux inclusions et au niveau de risque qui a été attribué à la recherche.

- Visite d‘ouverture du centre : avant inclusion, pour une mise en place du protocole et prise de connaissance avec les différents intervenants de la recherche biomédicale.

- Lors des visites suivantes, les cahiers d'observation seront revus au fur et à mesure de l'état d'avancement de la recherche par les ARC. L'investigateur principal du centre ainsi que les autres investigateurs qui incluent ou assurent le suivi des personnes participant à la recherche s’engagent à recevoir les ARC à intervalles réguliers.

Lors de ces visites sur site et en accord avec les Bonnes Pratiques Cliniques, les éléments suivants seront revus :

- Respect du protocole et des procédures définies pour la recherche,
- Vérification des consentements éclairés des patients
- Examen des documents source et confrontation avec les données reportées dans le cahier d’observation quant à l’exactitude, les données manquantes, la cohérence des données selon les règles édictées par les procédures du DRCD.

- Visite de fermeture : récupération des cahiers d’observation, bilan à la pharmacie, documents de la recherche biomédicale, archivage.

### b. Cahier d’observation

Le cahier d’observation comportera pour chaque patient à la visite de pré-screening (V0):

- L’identification du patient (numéro patient, la première lettre du nom et du prénom, sa date de naissance)
- La date de la visite
- Les critères de sélection et de non-inclusion
- Le taux d’IgE spécifiques au venin d’abeille
- Le résultat de l’intradermoréaction au venin d’abeille

Le cahier d’observation comportera pour chaque patient à la visite de sélection (V1):

- L’identification du patient (numéro patient, la première lettre du nom et du prénom, sa date de naissance)
- La date de la visite
- Un examen clinique général (le poids, la taille et les signes vitaux)
- Un bilan biologique (hémogramme, plaquettes, transaminases, ionogramme, créatininémie)
- Un ECG
- Les antécédents médicaux et chirurgicaux
- Les traitements en cours
- Mini Mental State (MMS)
- Batterie rapide d'évaluation frontale (BREF)
- UPDRS I-IV
- Echelle ségmentale
- Echelle Schwab & England
- Echelle de Hoehn et Yahr
- Date de l’examen SPECT

Le cahier d’observation comportera pour chaque patient aux visites de traitement (V2 – V14):

- L’identification du patient + le numéro de randomisation
- La date de la visite
- Le poids
- Les signes vitaux du patient avant et après injection du venin d’abeille/placebo
- Un bilan biologique (hémogramme, plaquettes, transaminases, ionogramme, créatininémie) et taux d'IgE spécifiques au venin d’abeille à V3, V5, V7, V9, V11 et V14
- Un ECG du sujet avant et après injection du venin d’abeille/placebo
- Les traitements nouveaux et en cours
- Mini Mental State (MMS – V14 uniquement)
- Batterie rapide d'évaluation frontale (BREF – V14 uniquement)
- Résultats du PDQ-39 (V2 et V14 uniquement)
- UPDRS I-IV
- Echelle de Hoehn et Yahr
- Date de l’examen SPECT (à V14 uniquement)
- Une fiche de survenue d’événements indésirables.

Toutes les informations requises par le protocole doivent être fournies dans le cahier d’observation et une explication donnée par l’investigateur pour chaque donnée manquante.

Les données devront être transférées dans les cahiers d'observation au fur et à mesure qu'elles sont obtenues. Les données devront être copiées de façon nette et lisible à l'encre noire dans ces cahiers (ceci afin de faciliter la duplication et la saisie informatique).

Les données erronées dépistées sur les cahiers d'observation seront clairement barrées et les nouvelles données seront copiées sur le cahier avec les initiales et la date par le membre de l'équipe de l'investigateur qui aura fait la correction.

L'anonymat des sujets sera assuré par la mention des initiales du sujet sur tous les documents nécessaires à la recherche, ou par effacement par les moyens appropriés (blanc correcteur…) des données nominatives sur les copies des documents source, destinés à la documentation de la recherche.

Les données informatisées sur un fichier seront déclarées à la CNIL.

## 14- Aspects légaux et éthiques

Le promoteur est défini par la loi 2004-806 du 9 août 2004. Dans cette recherche, l'AP-HP est le promoteur et le Département de la Recherche Clinique et du Développement (DRCD) en assure les missions réglementaires.

Avant de démarrer la recherche, chaque investigateur fournira au représentant du promoteur de la recherche une copie de son **curriculum vitæ personnel daté et signé** et comportant son numéro d’inscription à l’ordre des médecins.

**a. Demande d’autorisation auprès de l’Afssaps**

Pour pouvoir démarrer la recherche, l’AP-HP en tant que promoteur doit soumettre un dossier de demande d’autorisation auprès de l'autorité compétente l’Afssaps. L'autorité compétente, définie à l'article L. 1123-12, se prononce au regard de la sécurité des personnes qui se prêtent à une recherche biomédicale, en considérant notamment la sécurité et la qualité des produits utilisés au cours de la recherche conformément, le cas échéant, aux référentiels en vigueur, leur condition d'utilisation et la sécurité des personnes au regard des actes pratiqués et des méthodes utilisées ainsi que les modalités prévues pour le suivi des personnes.

**b. Demande d’avis au Comité de Protection des Personnes**

En accord avec l'article L.1123-6 du Code de Santé Publique, le protocole de recherche doit être soumis par le promoteur à un Comité de Protection des Personnes L'avis de ce comité est notifié à l’autorité compétente par le promoteur avant le démarrage de la recherche.

**c. Modifications**

Le DRCD doit être informé de tout projet de modification du protocole par l’investigateur coordonnateur. Les modifications devront être qualifiées en substantielles ou non.

Une modification substantielle est une modification susceptible, d'une manière ou d'une autre, de modifier les garanties apportées aux personnes qui se prêtent à la recherche biomédicale (modification d’un critère d’inclusion, prolongation d’une durée d’inclusion, participation de nouveaux centres,…).

Après le commencement de la recherche, toute modification substantielle de celle-ci à l’initiative du promoteur doit obtenir, préalablement à sa mise en oeuvre, un avis favorable du comité et une autorisation de l’autorité compétente. Dans ce cas, si cela est nécessaire, le comité s’assure qu’un nouveau consentement des personnes participant à la recherche est bien recueilli.

Par ailleurs, toute extension de la recherche (modification profonde du schéma thérapeutique ou des populations incluses, prolongation des traitements et ou des actes thérapeutiques non prévus initialement dans le protocole) devra être considérée comme une nouvelle recherche.

Toute modification substantielle devra faire l’objet **par le promoteur** d’une demande d’autorisation auprès de l’Afssaps et/ou d’une demande d’avis du CPP.

**d. Déclaration CNIL**

La loi prévoit que la déclaration du fichier informatisé des données personnelles collectées pour la recherche doit être faite avant le début effectif de la recherche.

Une déclaration normale à la CNIL sera faite avant le démarrage de l’étude.

### e. Note d’information et Consentement éclairé écrit

Le consentement écrit doit être recueilli auprès de toute personne se prêtant à la recherche avant la réalisation de tout acte nécessité par la recherche biomédicale.

L’investigateur s’assurera que le sujet a bien compris les implications de la participation à l’essai et recueillera son consentement écrit (loi N°88-138, Art L.209.9). L’investigateur laissera au patient un délai de réflexion éventuel entre l’information donnée par l’investigateur et la signature du consentement écrit.

Un double des documents de consentement sera remis à chaque patient.

**f. Rapport final de la recherche**

Le rapport final de la recherche sera écrit en collaboration par le coordonnateur et le biostatisticien pour cette recherche. Ce rapport sera soumis à chacun des investigateurs pour avis. Une fois qu'un consensus aura été obtenu, la version finale devra être avalisée par la signature de chacun des investigateurs et adressée au promoteur dans les meilleurs délais après la fin effective de la recherche. Un rapport rédigé selon le plan de référence de l’autorité compétente doit être transmis à l’autorité compétente ainsi qu’au CPP dans un délai de un an, après la fin de la recherche, s’entendant comme la dernière visite de suivi du dernier sujet inclus. Ce délai est rapporté à 90 jours en cas d’arrêt prématuré de la recherche

**15- Traitement des données et conservation des documents et des données relatives à la**

**recherche**

Les documents d’une recherche entrant dans le cadre de la loi sur les recherches biomédicales doivent être archivés par toutes les parties pendant une durée de 15 ans après la fin de la recherche.

cet archivage indexé comporte :

- Les copies de courrier d’autorisation de l’Afssaps et de l’avis obligatoire du CPP
- Les versions successives du protocole (identifiées par le n° de version et la date de version),
- Les courriers de correspondance avec le promoteur,
- Les consentements signés des sujets sous pli cacheté avec la liste ou registre d’inclusion en correspondance,
- Le cahier d’observation complété et validé de chaque sujet inclus,
- Toutes les annexes spécifiques à l’étude,
- Le rapport final de l’étude provenant de l’analyse statistique et du contrôle qualité de l’étude (double transmis au promoteur).
- Les certificats d’audit éventuels réalisés au cours de la recherche.

La base de données ayant donné lieu à l’analyse statistique doit aussi faire l’objet d’archivage par le responsable de l’analyse (support papier ou informatique).

**16- Assurance et engagement scientifique** *(éventuellement contrat ou convention si ces points*

*ne font pas l'objet d'un document distinct).*

*a. Assurance*

L'Assistance Publique- Hôpitaux de Paris est le promoteur de cette recherche. En accord avec la loi sur les recherches biomédicales, elle a pris une assurance auprès de la compagnie GERLING KonZern pour toute la durée de la recherche, garantissant sa propre responsabilité civile ainsi que celle de tout intervenant (médecin ou personnel impliqué dans la réalisation de la recherche) (loi n°2004-806, Art L.1121-10 du CSP).

l'Assistance Publique - Hôpitaux de Paris se réserve le droit d'interrompre la recherche à tout moment pour des raisons médicales ou administratives; dans cette éventualité, une notification sera fournie à l'investigateur.

**b. Engagement scientifique**

Chaque investigateur s'engagera à respecter les obligations de la loi et à mener la recherche selon les B.P.C., en respectant les termes de la déclaration d'Helsinki en vigueur. Pour ce faire, un exemplaire de **l’engagement scientifique (document type DRCD)** daté et signé **par chaque investigateur** de chaque service clinique d’un centre participant sera remis au représentant du promoteur.

**17- Règles relatives à la publication**

L’AP-HP est propriétaire des données et aucune utilisation ou transmission à un tiers ne peut être effectuée sans son accord préalable.

seront premiers signataires des publications, les personnes ayant réellement participé à l’élaboration du protocole et son déroulement ainsi qu’à la rédaction des résultats.

L’Assistance Publique- Hôpitaux de Paris doit être mentionnée comme étant le promoteur de la recherche biomédicale et comme soutien financier le cas échéant. les termes « Assistance Publique- Hôpitaux de Paris » doivent apparaître dans l’adresse des auteurs.

# 18 - Références

Ashcroft FM, Gribble FM. Correlating structure and function in ATP-sensitive K+ channels. Trends Neurosci. 1998 Jul;21(7):288-94.

el Mestikawy S, Gozlan H, Glowinski J, Hamon M. Characteristics of tyrosine hydroxylase activation by K+-induced depolarization and/or forskolin in rat striatal slices. J Neurochem. 1985 Jul;45(1):173-84.

Gandhi S, Wood NW. Molecular pathogenesis of Parkinson's disease. Hum Mol Genet. 2005 Sep 15;14(18):2749-55.

Gonon FG, Buda MJ. Regulation of dopamine release by impulse flow and by autoreceptors as studied by in vivo voltammetry in the rat striatum. Neuroscience. 1985 Mar;14(3):765-74.

Hirsch E, Graybiel AM, Agid YA. Melanized dopaminergic neurons are differentially susceptible to degeneration in Parkinson's disease. Nature. 1988 Jul 28;334(6180):345-8.

Liss B, Haeckel O, Wildmann J, Miki T, Seino S, Roeper J. K-ATP channels promote the differential degeneration of dopaminergic midbrain neurons. Nat Neurosci. 2005 Dec;8(12):1742-51.

Michel PP, Alvarez-Fischer D, Guerreiro S, Hild A, Hartmann A, Hirsch EC. Role of activity-dependent mechanisms in the control of dopaminergic neuron survival. J Neurochem. 2007 Apr;101(2):289-97.

Michel PP, Ruberg M, Hirsch E. Dopaminergic neurons reduced to silence by oxidative stress: an early step in the death cascade in Parkinson's disease? Sci STKE. 2006 Apr 25;2006(332):pe19.

Parkinson Study Group. Pramipexole vs levodopa as initial treatment for Parkinson disease: A randomized controlled trial. JAMA. 2000 Oct 18;284(15):1931-8.

Poyot T, Condé F, Grégoire MC, Frouin V, Coulon C, Fuseau C, Hinnen F, Dollé F, Hantraye P, Bottlaender M. Anatomic and biochemical correlates of the dopamine transporter ligand 11C-PE2I in normal and parkinsonian primates: comparison with 6-[18F]fluoro-L-dopa. J Cereb Blood Flow Metab. 2001 Jul;21(7):782-92.

Ribeiro MJ, Vidailhet M, Loc'h C, Dupel C, Nguyen JP, Ponchant M, Dollé F, Peschanski M, Hantraye P, Cesaro P, Samson Y, Remy P. Dopaminergic function and dopamine transporter binding assessed with positron emission tomography in Parkinson disease. Arch Neurol. 2002 Apr;59(4):580-6.

Salthun-Lassalle B, Hirsch EC, Wolfart J, Ruberg M, Michel PP. Rescue of mesencephalic dopaminergic neurons in culture by low-level stimulation of voltage-gated sodium channels. J Neurosci.2004 Jun 30;24(26):5922-30.

Salthun-Lassalle B, Traver S, Hirsch EC, Michel PP. Substance P, neurokinins A and B, and synthetic tachykinin peptides protect mesencephalic dopaminergic neurons in culture via an activity-dependent mechanism. Mol Pharmacol. 2005 Nov;68(5):1214-24.

Schapira AH. Mitochondria in the aetiology and pathogenesis of Parkinson's disease. Lancet Neurol. 2008 Jan;7(1):97-109.

Shults CW, Oakes D, Kieburtz K, Beal MF, Haas R, Plumb S, Juncos JL, Nutt J, Shoulson I, Carter J, Kompoliti K, Perlmutter JS, Reich S, Stern M, Watts RL, Kurlan R, Molho E, Harrison M, Lew M; Parkinson Study Group. Effects of coenzyme Q10 in early Parkinson disease: evidence of slowing of the functional decline. Arch Neurol. 2002 Oct;59(10):1541-50.

Tolosa E, Borght TV, Moreno E; DaTSCAN Clinically Uncertain Parkinsonian Syndromes Study GroupMembers of the DaTSCAN CUPS Study Group are listed as an Appendix. Accuracy of DaTSCAN ((123)I-ioflupane) SPECT in diagnosis of patients with clinically uncertain parkinsonism: 2-Year follow-up of an open-label study. Mov Disord. 2007 Dec 15;22(16):2346-51.

Waroux O, Massotte L, Alleva L, Graulich A, Thomas E, Liégeois JF, Scuvée-Moreau J, Seutin V. SK channels control the firing pattern of midbrain dopaminergic neurons in vivo. Eur J Neurosci.2005 Dec;22(12):3111-21.

Whone AL, Watts RL, Stoessl AJ, Davis M, Reske S, Nahmias C, Lang AE, Rascol O, Ribeiro MJ, Remy P, Poewe WH, Hauser RA, Brooks DJ; REAL-PET Study Group. Slower progression of Parkinson's disease with ropinirole versus levodopa: The REAL-PET study. Ann Neurol. 2003 Jul;54(1):93-101.

Whone AL, Bailey DL, Remy P, Pavese N, Brooks DJ. A technique for standardized central analysis of 6-(18)F-fluoro-L-DOPA PET data from a multicenter study. J Nucl Med. 2004 Jul;45(7):1135-45.

Wolfart J, Roeper J. Selective coupling of T-type calcium channels to SK potassium channels prevents intrinsic bursting in dopaminergic midbrain neurons. J Neurosci. 2002 May 1;22(9):3404-13.

**Annexe 1 : schéma de l’étude**

| **Mois** | **M-2** | **M - 1** | | **M0** | **M1 – M11** | **M12** |
| --- | --- | --- | --- | --- | --- | --- |
| **N° de la visite** | **V0** | **V1** | | **V2** | **V3 – V13** | **V14** |
| **Description** | Visite de pré-screening | Visite de sélection | | Visite de 1^ère^ injection | Visites de suivi | Visite de fin d’étude |
| **Contenu** |  | |  | | | |
| Signature du consentement | + |  | |  |  |  |
| Examen clinique, taille |  | + | |  |  | + |
| ECG |  | + | | + | + | + |
| Bilan sanguin |  | + | | + | + | + |
| Intradermoréaction au venin d’abeille | + |  | |  |  |  |
| Détermination des IgE spécifique au venin d’abeille | + |  | |  | + à V3, V5, V7, V9 et V11 | + |
| Poids et signes vitaux |  | + | | + | + | + |
| Vérification des co-traitements |  | + | | + | + | + |
| Vérification des antécédents médicaux et chirurgicaux |  | + | |  |  |  |
| MMS |  | + | |  |  | + |
| BREF |  | + | |  |  | + |
| PDQ-39 |  |  | | + |  | + |
| UPDRS I - IV |  | + | | + | + | + |
| Schwab & England |  | + | | + | + | + |
| Echelle ségmentale |  | + | | + | + | + |
| Vidéo UPDRS III |  |  | | + | + (V13 uniquement) |  |
| Recueil effets indésirables |  |  | | + | + | + |
| Hoehn & Yahr |  | + | | + | + | + |
| SPECT |  | + | |  |  | + |
| IRM |  | + | |  |  |  |
| Injection venin d’abeille/placebo |  |  | | + | + |  |

# Annexe 2 : score UPDRS

**UPDRS I**

État mental, comportemental et thymique

**1. Affaiblissement intellectuel : /4**

0 = Absent.

1 = Léger. Manque de mémoire habituel avec souvenir partiel des événements sans autre difficulté.

2 = Perte mnésique modérée, avec désorientation et difficultés modérées à faire face à des problèmes complexes. Atteinte légère mais indiscutable de ses capacités fonctionnelles avec besoin d’une incitation occasionnelle de l’entourage.

3 = Déficit mnésique grave avec désorientation dans le temps et souvent dans l’espace. Handicap grave face aux problèmes.

4 = Perte mnésique sévère avec uniquement conservation de sa propre orientation. Incapable de porter des jugements ou de résoudre des problèmes, demande beaucoup d’aide pour les soins personnels, ne peut plus être laissé seul.

**2. Troubles de la pensée : /4**

0 = Aucun.

1 = Rêves animés.

2 = Hallucinations bénignes critiquées.

3 = Hallucinations occasionnelles ou fréquentes ou idées délirantes non critiquées : peuvent gêner les

activités quotidiennes.

4 = Hallucinations continuelles. Idées délirantes ou psychose expansive : incapable de prendre soin de

lui-même.

**3. Dépression : /4**

0 = Absente.

1 = Périodes de tristesse ou sentiment de culpabilité excessif ne persistant pas plusieurs jours ou semaines.

2 = Dépression durable avec symptômes végétatifs (insomnie, anorexie, perte de poids, perte d’intérêt).

4 = Dépression durable avec symptômes végétatifs et pensées ou intentions suicidaires.

**4. Motivation - Initiative : /4**

0 = Normale.

1 = Moins franche qu’à l’habitude : plus passif.

2 = Perte d’initiative avec désintérêt pour certaines activités non routinières.

3 = Perte d’initiative ou désintérêt dans les activités quotidiennes routinières.

4 = Absence d’initiative, perte totale d’intérêt.

**UPDRS II**

Activités de la vie quotidienne

**5. Parole : On : /4 Off : /4**

0 = Normale.

1 = Légèrement perturbée, pas de difficultés à être compris.

2 = Modérément perturbée. On doit occasionnellement lui demander de répéter.

3 = Gravement perturbée. On doit lui demander fréquemment de répéter.

4 = Incompréhensible la plupart du temps.

**6. Salivation : On : /4 Off : /4**

0 = Normale.

1 = Légère mais excès habituel de salive dans la bouche, peut baver pendant la nuit.

2 = Hypersialorrhée modérée. Peut baver un peu.

3 = Hypersialorrhée nette avec un peu de bave.

4 = Écoulement habituel de bave nécessitant en permanence un mouchoir.

**7. Déglutition : On : /4 Off : /4**

0 = Normale.

1 = S’étrangle rarement.

2 = S’étrangle occasionnellement.

3 = Nécessite une alimentation semi-liquide.

4 = Nécessite une alimentation par sonde gastrique ou une gastrostomie.

**8. Écriture : On : /4 Off : /4**

0 = Normale.

1 = Légèrement ralentie ou micrographique.

2 = Nettement ralentie ou micrographique, tous les mots sont lisibles.

3 = Gravement perturbée : tous les mots ne sont pas lisibles.

4 = La majorité des mots est illisible.

**9. S’alimenter et manipuler les couverts : On : /4 Off : /4**

0 = Normal.

1 = Un peu lent et maladroit, mais n’a pas besoin d’être aidé.

2 = Pour la plupart des aliments, peut se débrouiller seul quoique maladroit et lent.

3 = A besoin d’une aide pour les repas mais peut encore s’alimenter lentement.

4 = On doit lui donner à manger.

**10. Habillage : On : /4 Off : /4**

0 = Normal.

1 = Un peu lent, mais ne doit pas être aidé.

2 = Aide occasionnelle pour boutonner, enfiler une manche.

3 = A besoin d’être très aidé mais peut encore faire certaines choses seul.

4 = Totalement dépendant.

**11. Hygiène : On : /4 Off : /4**

0 = Normale.

1 = Un peu lent mais n’a pas besoin d’être aidé.

2 = Nécessite une aide pour la douche et le bain, ou très lent dans les soins hygiéniques.

3 = Nécessite une aide pour se laver, se brosser les dents, se coiffer et se baigner.

4 = Sonde urinaire ou autres aides mécaniques.

**12. Se retourner dans le lit : On : /4 Off : /4**

0 = Normal.

1 = Un peu lent et maladroit, mais n’a pas besoin d’être aidé.

2 = Peut se retourner seul ou arranger les draps mais avec une grande difficulté.

3 = Peut commencer le geste mais n’arrive pas à se retourner ou arranger les draps seul.

4 = Dépendant.

**13. Chute non liée au piétinement : On : /4 Off : /4**

0 = Aucune.

1 = Chutes rares.

2 = Chutes occasionnelles mais moins d’une fois par jour.

3 = En moyenne, une chute par jour.

4 = Chutes pluriquotidiennes.

**14. Piétinement lors de la marche : On : /4 Off : /4**

0 = Aucun. 1 = Rare piétinement lors de la marche, peut avoir une hésitation au départ.

2 = Piétinement occasionnel lors de la marche.

3 = Piétinement fréquent entraînant occasionnellement des chutes.

4 = Chutes fréquentes dues aux piétinements.

**15. Marche : On : /4 Off : /4**

0 = Normale.

1 = Difficultés légères, mais peut balancer les bras ou traîner les pieds.

2 = Difficultés modérées mais ne demande que peu ou pas d’aide.

3 = Difficultés importantes à la marche nécessitant une aide.

4 = Ne peut pas marcher du tout, même avec une aide.

**16. Tremblement : On : /4 Off : /4**

0 = Absent.

1 = Léger et rarement présent.

2 = Modéré, gênant le patient.

3 = Important, gêne certaines activités.

4 = Marqué, gêne la plupart des activités.

**17. Troubles sensitifs subjectifs liés au parkinsonisme : On : /4 Off : /4**

0 = Aucun.

1 = Occasionnellement engourdissements, picotements ou douleurs légères.

2 = Engourdissements, picotements ou douleurs fréquentes : pas gênant.

3 = Sensations douloureuses fréquentes.

4 = Douleurs très vives.

**UPDRS III**

Examen moteur

**18. Parole : /4**

0 = Normale.

1 = Légère perte d’expression, de la diction et/ou du volume vocal.

2 = Voix monotone, bredouillée mais compréhensible, altération modérée.

3 = Altération marquée, difficile à comprendre.

4 = Incompréhensible

**19. Expression faciale : /4**

0 = Normale.

1 = Hypomimie légère, semble avoir un visage normalement impassible.

2 = Diminution légère mais franchement anormale de l’expression faciale.

3 = Hypomimie modérée : lèvres souvent entrouvertes.

4 = Masque facial ou faciès figé avec perte importante ou totale de l’expression faciale : lèvres entrouvertes (0,6 cm ou plus).

**20. Tremblement de repos :**

Menton - Lèvres /4

Bras D : /4 G : /4

Jambes D : /4 G : /4

0 = Absent.

1 = Léger et rarement présent.

2 = Tremblement de faible amplitude mais persistant, ou d’amplitude modérée, mais présent seulement de façon intermitente.

3 = Tremblement modéré en amplitude et présent la plupart du temps.

4 = Tremblement d’amplitude marquée et présent la plupart du temps.

**21. Tremblement d’action ou postural des mains : D : /4 G : /4**

0 = Absent.

1 = Léger : présent lors de l’action.

2 = Modéré en amplitude, présent lors de l’action.

3 = Modéré en amplitude, tant lors du maintien postural que lors de l’action.

4 = Amplitude marquée : gêne l’alimentation.

**22. Rigidité :**

Cou /4

Bras D : /4 G : /4

Jambes D : /4 G : /4

0 = Absente.

1 = Minime ou apparaissant lors des manoeuvres de sensibilisation.

2 = Légère à modérée.

3 = Marquée, mais la plupart des mouvements peuvent être effectués aisément.

4 = Sévère, les mouvements sont effectués difficilement.

**23. Tapotement des doigts (Pince pouce / index) : D : /4 G : /4**

0 = Normal.

1 = Ralentissement léger et/ou réduction d’amplitude.

2 = Modérément perturbé, se fatigue nettement et rapidement, peut avoir d’occasionnels arrêts du mouvement.

3 = Sévèrement perturbé. Hésitations fréquentes au démarrage du mouvement.

4 = Peut à peine effectuer le mouvement.

**24. Mouvements des mains (Ouverture / fermeture) : D : /4 G : /4**

0 = Normaux.

1 = Ralentissement léger et/ou réduction d’amplitude.

2 = Modérément perturbés. Se fatigue nettement et rapidement, peut avoir d’occasionnels arrêts dans

le mouvement.

3 = Sévèrement perturbés, hésitation fréquente au début du mouvement ou arrêt en cours de

mouvement.

4 = Peut à peine effectuer la tâche.

**25. Mouvements alternatifs rapides (Marionnettes) : D : /4 G : /4**

0 = Normaux.

1 = Ralentissement léger et/ou réduction d’amplitude.

2 = Modérément perturbés. Se fatigue nettement et rapidement, peut avoir d’occasionnels arrêts dans

le mouvement.

3 = Sévèrement perturbés, hésitation fréquente au début du mouvement ou arrêt en cours de

mouvement.

4 = Peut à peine effectuer la tâche.

**26. Agilité de la jambe : D : /4 G : /4**

0 = Normale.

1 = Ralentissement léger et/ou réduction d’amplitude.

2 = Modérément perturbée. Se fatigue nettement et rapidement, peut avoir d’occasionnels arrêts dans

le mouvement.

3 = Sévèrement perturbée, hésitation fréquente au début du mouvement ou arrêt en cours de

mouvement.

4 = Peut à peine effectuer la tâche.

**27. Se lever d’une chaise : /4**

0 = Normal.

1 = Lentement ou a besoin de plus d’un essai.

2 = Pousse sur les bras du siège.

3 = Tend à tomber en arrière et doit essayer plus d’une fois mais peut se lever sans aide.

4 = Incapable de se lever sans aide.

**28. Posture : /4**

0 = Normalement droite.

1 = Pas tout à fait droite, posture légèrement fléchie : cette attitude peut être normale pour une

personne plus âgée.

2 = Posture modérément fléchie, nettement anormale : peut être légèrement penché d’un côté.

3 = Posture sévèrement fléchie avec cyphose : peut être modérément penché d’un côté.

4 = Flexion marquée avec posture très anormale.

**29. Stabilité posturale : /4**

0 = Normale.

1 = Rétropulsion mais rétablit l’équilibre sans aide.

2 = Absence de réponse posturale : peut tomber s’il n’est pas retenu par l’examinateur.

3 = Très instable, tend à perdre l’équilibre spontanément.

4 = Incapable de se tenir debout sans aide.

**30. Démarche : /4**

0 = Normale.

1 = Marche lentement, mais traîne les pieds et fait de petits pas, mais sans festination, ni propulsion.

2 = Marche avec difficulté, mais nécessite peu ou pas d’aide : festination, petits pas ou propulsion

possibles.

3 = Perturbation sévère de la marche, nécessitant une aide.

4 = Ne peut pas marcher du tout, même avec une aide.

**31. Bradykinésie corporelle et hypokinésie : /4**

0 = Aucune.

1 = Lenteur minime, donnant aux mouvements un caractère délibéré, pourrait être normal pour certaines personnes.

Possibilité d’une réduction d’amplitude.

2 = Degré léger de lenteur et de pauvreté du mouvement qui est nettement anormal. De plus, il existe

une certaine réduction d’amplitude.

3 = Lenteur modérée, pauvreté et petite amplitude du mouvement.

4 = Lenteur marquée, pauvreté et petite amplitude du mouvement.

TOTAL sur 108

**UPDRS IV**

Complications du traitement (au cours de la dernière semaine)

A) DYSKINESIES

**32. Durée**

0 = Aucune

1 = 1 à 25% de la journée

2 = 26 à 50% de la journée

3 = 51 à 75% de la journée

4 = 76 à 100% de la journée

**33. Incapacité**

0 = Aucune

1 = Légère

2 = Modérée

3 = Sévère

4 = Complète

**34. Dyskinésies douloureuses**

0 = Aucune

1 = Légères

2 = Modérées

3 = Sévères

4 = Marquées

**35 Présence d’une dystonie matinale**

oui = 1

non = 0

B) FLUCTUATIONS CLINIQUES

**36. Périodes OFF prédictives**

oui = 1

non = 0

**37. Périodes OFF non prédictives**

oui = 1

non = 0

**38. Périodes OFF brutales**

oui = 1

non = 0

**39. Proportion de OFF**

1 = 1 à 25% de la journée

2 = 26 à 50% de la journée

3 = 51 à 75% de la journée

4 = 76 à 100% de la journée

C) AUTRES COMPLICATIONS

**40. anorexie, nausées, vomissements**

oui = 1

non = 0

**41. insomnies, somnolence**

oui = 1

non = 0

**42. hypotension orthostatique**

oui = 1

non = 0

TOTAL sur 23

**Annexe 3 : Stade de Hoehn et Yahr**

Stade 0 : pas de signe de la maladie.

Stade 1 : maladie unilatérale.

Stade 1,5 : maladie unilatérale, plus atteinte axiale.

Stade 2 : maladie bilatérale sans troubles de l’équilibre.

Stade 2,5 : maladie bilatérale légère avec rétablissement lors du test de la poussée.

Stade 3 : maladie bilatérale légère à modérée : une certaine instabilité posturale, physiquement autonome.

Stade 4 : handicap sévère : toujours capable de marcher ou de se tenir debout sans aide.

Stade 5 : malade en chaise roulante ou alité sauf s’il est aidé.

# Annexe 4 : Extrait du VIDAL 2009 concernant le médicament utilisé

**VIDAL 2009**

**Médicaments**

*** ALYOSTAL VENINS   STALLERGÈNES®**

**abeille Apis mellifera, guêpes Vespula, guêpes Polistes**

[Formes et présentations](../../../../Users/andreas/Library/Preferences/VIDALexpert/Temp/3328267.html#forme) | [Composition](../../../../Users/andreas/Library/Preferences/VIDALexpert/Temp/3328267.html#compo) | [Indications](../../../../Users/andreas/Library/Preferences/VIDALexpert/Temp/3328267.html#indic) | [Posologie et mode d'administration](../../../../Users/andreas/Library/Preferences/VIDALexpert/Temp/3328267.html#posol) | [Contre-indications](../../../../Users/andreas/Library/Preferences/VIDALexpert/Temp/3328267.html#contr) | [Mises en garde et précautions d'emploi](../../../../Users/andreas/Library/Preferences/VIDALexpert/Temp/3328267.html#mises) | [Grossesse et allaitement](../../../../Users/andreas/Library/Preferences/VIDALexpert/Temp/3328267.html#gross) | [Effets indésirables](../../../../Users/andreas/Library/Preferences/VIDALexpert/Temp/3328267.html#effet) | [Surdosage](../../../../Users/andreas/Library/Preferences/VIDALexpert/Temp/3328267.html#surdo) | [Pharmacodynamie](../../../../Users/andreas/Library/Preferences/VIDALexpert/Temp/3328267.html#phard) | [Conditions de conservation](../../../../Users/andreas/Library/Preferences/VIDALexpert/Temp/3328267.html#condi) | [Prescription/délivrance/prise en charge](../../../../Users/andreas/Library/Preferences/VIDALexpert/Temp/3328267.html#rensadm)

| **FORMES et PRÉSENTATIONS** [(début page)](../../../../Users/andreas/Library/Preferences/VIDALexpert/Temp/3328267.html#pub) |
| --- |

*Poudre et solvant pour solution injectable (SC) à 110 µg et à 550 µg :* Flacon de poudre et flacon de solvant.

| **COMPOSITION** [(début page)](../../../../Users/andreas/Library/Preferences/VIDALexpert/Temp/3328267.html#pub) | |
| --- | --- |
| *Poudre à 110 µg :* | *p flacon* |
| Venin^*^ | 110 µg |

*Excipients :* albumine humaine, mannitol.

Flacon contenant 100 µg de venin par ml après reconstitution par 1,1 ml de solvant.

| *Poudre à 550 µg :* | *p flacon* |
| --- | --- |
| Venin^*^ | 550 µg |

*Excipients :* albumine humaine, mannitol.

Flacon contenant 100 µg de venin par ml après reconstitution par 5,5 ml de solvant.

^*^ Abeille Apis mellifera ou guêpes Vespula ou guêpes Polistes. Les principaux allergènes protéiques du venin d'abeille sont la phospholipase A2, la hyaluronidase et la phosphatase acide. Les peptides sont la mellitine, l'apamine, et le mastocyte MDC peptide.

Venin de guêpes Vespula provenant d'un mélange de plusieurs espèces (composition en protéines : phospholipase A_1_, hyaluronidase, antigène 5 phosphatase acide ; les peptides sont le mastocyte MDC peptide, kinine).

Venin de guêpes Polistes provenant d'un mélange de venins de différentes espèces de guêpes Polistes dont les 3 principales protéines sont l'antigène 5, la hyaluronidase et la phospholipase A_1_.

| DC | **INDICATIONS** [(début page)](../../../../Users/andreas/Library/Preferences/VIDALexpert/Temp/3328267.html#pub) |
| --- | --- |

1. Diagnostic cutané des allergies aux piqûres de l'hyménoptère identifié.
2. Hyposensibilisation par immunothérapie spécifique des manifestations d'allergie au venin d'hyménoptères identifié.

| DC | **POSOLOGIE ET MODE D'ADMINISTRATION** [(début page)](../../../../Users/andreas/Library/Preferences/VIDALexpert/Temp/3328267.html#pub) |
| --- | --- |

**Posologie :**

Tableau donné à titre purement indicatif, le schéma thérapeutique devant être impérativement établi par l'allergologue qui a posé l'indication de l'hyposensibilisation spécifique.

1. Alyostal Venin ST peut être injecté à doses progressives par voie sous-cutanée jusqu'à la dose maximale bien tolérée (maximum 100 µg).
2. La concentration de départ sera 10 fois inférieure à la concentration « seuil de réactivité », ayant donné une IDR positive (dans le cas où elle a été effectuée).
3. La progression des doses, le volume de produit injecté ainsi que la fréquence des injections sont fonction de la sensibilité et du contexte d'utilisation du produit.
4. Le schéma par rush-désensibilisation est laissé à la seule initiative de l'allergologue et doit être appliqué en milieu hospitalier.

Tableau de traitement :

| Concentration | Volume   injecté | Intervalle | Conservation |
| --- | --- | --- | --- |
| 0,000001 µg/ml (10^-6^) | 0,1 ml | 2 injections   par   semaine | A préparer   extemporanément |
| 0,00001 µg/ml (10^-5^) | 0,1 ml |  |  |
| 0,0001 µg/ml (10^-4^) | 0,1 ml |  |  |
| 0,001 µg/ml (10^-3^) | 0,1 ml |  |  |
| 0,01 µg/ml (10^-2^) | 0,1 ml |  |  |
| 0,1 µg/ml (10^-1^) | 0,1 ml |  |  |
| 1 µg/ml | 0,1 ml |  |  |
|  | 0,2 ml |  |  |
|  | 0,4 ml |  |  |
|  | 0,8 ml |  |  |
| 10 µg/ml | 0,1 ml | 1 injection   tous les   4 à 6 jours |  |
|  | 0,2 ml |  |  |
|  | 0,4 ml |  |  |
|  | 0,8 ml |  |  |
| 100 µg/ml | 0,1 ml |  | Environ   6 mois |
|  | 0,2 ml |  |  |
|  | 0,3 ml |  |  |
|  | 0,4 ml |  |  |
|  | 0,5 ml |  |  |
|  | 0,6 ml |  |  |
|  | 0,7 ml |  |  |
|  | 0,8 ml |  |  |
|  | 0,9 ml |  |  |
|  | 1,0 ml |  |  |

**Mode d'administration :**

1. Nettoyer la peau et le bouchon perforable du flacon à l'alcool à 70^o^.
2. Pour les venins à 110 µg, prélever 1,1 ml de solvant et pour les venins à 550 µg, prélever 5,5 ml de solvant avec une seringue graduée au 1/100 munie d'une aiguille 15-5/10 et remettre en solution la poudre contenant le venin. L'excès de solvant doit être jeté. Agiter doucement par un mouvement de rotation pendant quelques minutes en évitant la formation de mousse.
3. Ponctionner et mesurer avec précision la dose à injecter.
4. Injecter par voie sous-cutanée à la face externe du bras ou dans la région deltoïdienne, en prenant soin de ne pas faire une injection intraveineuse (procéder à cet effet à une légère aspiration préalable).
5. Garder le patient sous observation une demi-heure après chaque injection.

| DC | **CONTRE-INDICATIONS** [(début page)](../../../../Users/andreas/Library/Preferences/VIDALexpert/Temp/3328267.html#pub) |
| --- | --- |

Cachexie, affections malignes, néphropathies, vascularite systémique (dont périartérite noueuse), déficience immunitaire grave, phase aiguë d'un état morbide quelconque.

Traitement par immunosuppresseurs ou corticoïdes, traitement par les bêtabloquants.

En cas de poussée fébrile, de crise d'asthme ou d'asthme mal stabilisé, l'injection ne sera faite qu'après le retour à la normale.

| DC | **MISES EN GARDE et PRÉCAUTIONS D'EMPLOI** [(début page)](../../../../Users/andreas/Library/Preferences/VIDALexpert/Temp/3328267.html#pub) |
| --- | --- |

**Mises en garde :**

1. Les tests ne peuvent être faits que par des spécialistes très entraînés en raison du risque de réactions systémiques qui doivent être traitées d'urgence.
2. La mise en route du traitement ne peut être faite que par des spécialistes et dans un milieu offrant toutes les conditions de sécurité nécessaires (milieu hospitalier avec possibilité de soins intensifs).

**Précautions d'emploi :**

1. Ne jamais injecter par voie intraveineuse (aspirer par rétroaction du piston avant chaque injection pour vérifier l'absence de sang dans la seringue).
2. Adapter la posologie à la sensibilité du malade (dose et dilution).
3. Respecter la progression prescrite par le spécialiste. La réduire si une réaction locale importante est constatée. Revenir au besoin à la dilution précédente.
4. Demander l'avis du spécialiste chaque fois que la poursuite du traitement pose un problème.
5. Conserver le malade sous surveillance directe une demi-heure après chaque injection.
6. Éviter un repas copieux le jour de l'injection.
7. Éviter un effort physique violent le même jour.
8. Chez les patients très sensibles présentant des réactions locales gênantes, administrer un antihistaminique 1 à 2 h avant l'injection.
9. Avoir toujours à portée de main : adrénaline injectable, corticoïde injectable, antihistaminique injectable (cf Effets indésirables).

| DC | **GROSSESSE et ALLAITEMENT** [(début page)](../../../../Users/andreas/Library/Preferences/VIDALexpert/Temp/3328267.html#pub) |
| --- | --- |

**Grossesse :**

Éviter d'entreprendre un test ou une cure d'hyposensibilisation au cours de la grossesse.

| DC | **EFFETS INDÉSIRABLES** [(début page)](../../../../Users/andreas/Library/Preferences/VIDALexpert/Temp/3328267.html#pub) |
| --- | --- |

Les réactions locales (érythème, prurit, oedème local) disparaissent dans les 48 heures.

Les réactions systémiques accompagnées d'hyperthermie modérée et d'asthénie sont peu fréquentes et généralement sans gravité :

1. si elles se manifestent, reprendre le traitement à la dose antérieure bien tolérée, augmenter ensuite progressivement les doses ;
2. en cas de réactions répétées, ne pas dépasser la dose bien tolérée.

*Choc anaphylactique :*

Bien qu'exceptionnel, sa particulière gravité doit demeurer toujours à l'esprit lorsqu'on pratique une injection d'extrait de venin d'hyménoptères.

1. Pratiquer immédiatement une injection SC ou IM de 0,25 à 1 ml d'adrénaline à 1/1000 (et éventuellement une injection IV ou IM d'une ampoule d'un corticoïde soluble injectable).
2. Les traitements symptomatiques par antihistaminique ou corticoïde injectable ne doivent pas retarder l'injection d'adrénaline en cas de choc anaphylactique.

| DC | **SURDOSAGE** [(début page)](../../../../Users/andreas/Library/Preferences/VIDALexpert/Temp/3328267.html#pub) |
| --- | --- |

En cas de réaction immédiate que peut exceptionnellement déterminer un surdosage accidentel, il faut pratiquer rapidement :

1. une injection SC de 0,25 à 1 ml d'adrénaline à 1/1000 ;
2. et éventuellement une injection IV ou IM d'une ampoule d'un corticoïde soluble injectable.

Les traitements symptomatiques par antihistaminique ou corticoïde injectable ne doivent pas retarder l'injection d'adrénaline en cas de choc anaphylactique.

| PP | **PHARMACODYNAMIE** [(début page)](../../../../Users/andreas/Library/Preferences/VIDALexpert/Temp/3328267.html#pub) |
| --- | --- |

Les venins d'hyménoptères injectés par voie sous-cutanée favorisent la synthèse des anticorps IgG circulants, dits bloquants, lesquels interceptent les allergènes avant que ces derniers puissent atteindre les IgE fixées sur les basophiles et les mastocytes et qui, de plus, les protègent contre les facteurs de dégranulation.

Les injections répétées de venin parviennent à empêcher progressivement la fabrication excessive des anticorps IgE, probablement par stimulation des lymphocytes T suppresseurs, véritables freins dont on connaît la défaillance au cours de l'allergie.

| DP | **CONDITIONS DE CONSERVATION** [(début page)](../../../../Users/andreas/Library/Preferences/VIDALexpert/Temp/3328267.html#pub) |
| --- | --- |

A conserver entre + 2 °C et + 8 °C.

| **PRESCRIPTION/DÉLIVRANCE/PRISE EN CHARGE** [(début page)](../../../../Users/andreas/Library/Preferences/VIDALexpert/Temp/3328267.html#pub) |
| --- |

LISTE I

| AMM | 332 826.7 (1990 rév 14. 06. 00) abeille Apis mellifera 110 µg. |
| --- | --- |
|  | 332 829.6 (1990 rév 20. 09. 00) abeille Apis mellifera 550 µg. |
|  | 332 825.0 (1990 rév 25. 04. 00) guêpes Vespula 110 µg. |
|  | 332 827.3 (1990 rév 20. 09. 00) guêpes Vespula 550 µg. |
|  | 332 830.4 (1990 rév 25. 04. 00) guêpes Polistes 110 µg. |
|  | 332 831.0 (1990 rév 20. 09. 00) guêpes Polistes 550 µg. |
|  |  |
| **PRIX indicatif :** | 17.67 euros (abeille Apis mellifera 110 µg). |
|  | 21.58 euros (guêpes Vespula 110 µg). |
|  | 26.76 euros (guêpes Polistes 110 µg). |
| Remb Séc soc à 65 %. Collect. | |
| Présentation à 550 µg : Collect. AP. | |

**STALLERGÈNES SA**

6, rue Alexis-de-Tocqueville. 92183 Antony cdx

Tél : 01 55 59 20 00. Fax : 01 55 59 20 01

## Annexe 5 : Carte Patient

**CARTE PATIENT**

***Merci de garder cette carte en permanence avec vous***

**Nom :_______________________________________ Prénom :__________________________________**

**Numéro de patient : ____________________**

Je participe à la recherche biomédicale « **Evaluation des effets symptomatiques et neuroprotecteurs du venin d’abeille dans le traitement de la maladie de Parkinson : essai randomisé en double aveugle versus placebo : ETUDE MIREILLE»,** dont le promoteur est l’AP-HP.

Je reçois le traitement suivant : ALYOSTAL® 100 µg venin d'abeille ou Placebo

**Date de début du traitement : ……./…..../……......**

**Durée de traitement : 12 mois maximum en double aveugle**

**Durée de l’essai : 13 mois**

**Je suis suivi(e) par le Dr_________________________________________**

**A l’Hôpital Pitié-Salpêtrière, Paris 75013**

**Tel. :** 01 42 16 18 16 / 06 74 35 17 96.

**Dates des visites de suivi prévues :**

Date de la réalisation du SPECT M-1 : : ……./…..../……......

Visite M0 : **……./…..../……......**

Visite M1 : **……./…..../……......**

Visite M2 : **……./…..../…….....**

Visite M3 : **……./…..../…….....**

Visite M4 : **……./…..../……......**

Visite M5 : **……./…..../……......**

Visite M6 : **……./…..../…….....**

Visite M7 : **……./…..../…….....**

Visite M8 : **……./…..../……......**

Visite M9 : **……./…..../……......**

Visite M10 : **……./…..../…….....**

Visite M11 : **……./…..../……......**

Visite M12 : **……./…..../……...... Date de la réalisation du SPECT M12 :** : **……./…..../……......**

**Centre antipoison Fernand Widal au 01. 40. 05. 48. 48**

**Annexe 6 : Mini Mental State Examination (MMSE) (Version consensuelle du GRECO)**

**Orientation_____________________________________________________________ / 10**

Je vais vous poser quelques questions pour apprécier comment fonctionne votre mémoire.

Les unes sont très simples, les autres un peu moins. Vous devez répondre du mieux que vous pouvez.

Quelle est la date complète d’aujourd’hui ?___________________________

Si la réponse est incorrecte ou incomplète, posées les questions restées sans réponse, dans l’ordre suivant :(0ou1)

1. En quelle année sommes-nous ?

2. En quelle saison ?

3. En quel mois ?

4. Quel jour du mois ?

5. Quel jour de la semaine ?

Je vais vous poser maintenant quelques questions sur l’endroit où nous trouvons. (0ou1)

6. Quel est le nom de l’hôpital où nous sommes ?

7. Dans quelle ville se trouve-t-il ?

8. Quel est le nom du département dans lequel est située cette ville ?

9. Dans quelle province ou région est située ce département ?

10. A quel étage sommes-nous ?

**Apprentissage___________________________________________________________ / 3**

Je vais vous dire trois mots ; je vous voudrais que vous me les répétiez et que vous essayiez de les retenir

car je vous les redemanderai tout à l’heure. (0ou1)

11. Cigare Citron Fauteuil

12. Fleur ou Clé ou Tulipe

13. Porte Ballon Canard

Répéter les 3 mots

**Attention et calcul / 5**

Voulez-vous compter à partir de 100 en retirant 7 à chaque fois ? (0ou1)

14. 93

15. 86

16. 79

17. 72

18. 65

Pour tous les sujets, même pour ceux qui ont obtenu le maximum de points, demander :

Voulez-vous épeler le mot MONDE à l’envers ?

**Rappel / 3**

Pouvez-vous me dire quels étaient les 3 mots que je vous ai demandés de répéter et de retenir tout à l’heure ?

(0ou1)

11. Cigare Citron Fauteuil

12. Fleur ou Clé ou Tulipe

13. Porte Ballon Canard

**Langage /8**

(0ou1)

Montrer un crayon. 22. Quel est le nom de cet objet ?

Montrer votre montre. 23. Quel est le nom de cet objet ?

24. Ecoutez bien et répétez après moi : « PAS DE MAIS, DE SI, NI DE ET »

Poser une feuille de papier sur le bureau, la montrer au sujet en lui disant : « Ecoutez bien et faites

ce que je vais vous dire :

25. Prenez cette feuille de papier avec votre main droite,

26. Pliez-la en deux,

27. Et jetez-la par terre. »

Tendre au sujet une feuille de papier sur laquelle est écrit en gros caractère : « FERMEZ LES YEUX »

et dire au sujet :

28. « Faites ce qui est écrit ».

Tendre au sujet une feuille de papier et un stylo, en disant :

29. « Voulez-vous m’écrire une phrase, ce que vous voulez, mais une phrase entière. »

**Praxies constructives /1**

Tendre au sujet une feuille de papier et lui demander : (0ou1)

30. « Voulez-vous recopier ce dessin ? »

**Score total: I__I__I/ 30**

**Annexe 7 : Echelle de SCHWAB et ENGLAND**

100% - totalement indépendant

90% - indépendant mais plus lent

80% - indépendant conscient de sa lenteur

70% - pas tout à fait indépendant (3 à 4 fois + lent)

60% - partiellement dépendant

50% - aidé dans 50% des activités

40 % - très dépendant

30% - peu d’activités effectuées seul

20% - ne fait rien seul – aidé légèrement

10 % - alité – totalement dépendant

0 % : alité – troubles végétatifs

# Annexe 8 : ECHELLE SEGMENTALE

**Tremblement**: le patient est

- assis pour la cotation des membres supérieurs,
- allongé pour la cotation des membres inférieurs

au repos lors d’un effort mental

L’effort mental consiste en une tâche mathématique (soustraction réitérative de 100) et ensuite de nommer des capitales ou des chefs d’états

4 bras proximal D/G

6 doigts D/G

a pouce

b dig.II

c dig.III

d dig.IV

e dig.V

3 cou

Quantification du tremblement

A) aspect temporel

Présence du tremblement sans prise en compte de la sévérité, seulement au repos,

information de l’anamnèse:

- 0 : absent
- 1 : jamais présent au repos complet, mais apparaît lors des manœuvres de provocation (concentration, tâches multiples, émotions fortes, fièvre etc.)
- 2 : présent de manière intermittente au repos, < 1h/jour
- 3 : présent de manière intermittente au repos, > 1h/jour, < moitié de la journée
- 4 : présent de manière intermittente au repos, > moitié de la journée
- 5 : présent toute la journée

4 bras proximal D/G

6 doigts D/G

a pouce

b dig.II

c dig.III

d dig.IV

e dig.V

3 cou

Quantification du tremblement

Amplitude maximale du tremblement au repos, lors d’un effort mental, attitude et action

- 0 : absent
- 1 : minime : amplitude < 0.5 cm, i.e. tremblement tout juste visible
- 2 : léger : amplitude < 1cm, i.e. bien visible, mais discret
- 3 : modéré : amplitude > 1cm
- 4 : sévère : amplitude ≥ 5 cm (pour la langue, le visage, les doigts: ≥ 2 cm

Tremblement d’attitude

- membre supérieur droit

distal absent ⬜ présent ⬜ 0 ⬜ 1 ⬜ 2 ⬜ 3 ⬜ 4

proximal absent ⬜ présent ⬜ 0 ⬜ 1 ⬜ 2 ⬜ 3 ⬜ 4

- membre supérieur gauche

distal absent ⬜ présent ⬜ 0 ⬜ 1 ⬜ 2 ⬜ 3 ⬜ 4

proximal absent ⬜ présent ⬜ 0 ⬜ 1 ⬜ 2 ⬜ 3 ⬜ 4

- membre inférieur droit

absent ⬜ présent ⬜ 0 ⬜ 1 ⬜ 2 ⬜ 3 ⬜ 4

- membre inférieur gauche

absent ⬜ présent ⬜ 0 ⬜ 1 ⬜ 2 ⬜ 3 ⬜ 4

Tremblement d’action

- membre supérieur droit

absent ⬜ présent ⬜ 0 ⬜ 1 ⬜ 2 ⬜ 3 ⬜ 4

- membre supérieur gauche

absent ⬜ présent ⬜ 0 ⬜ 1 ⬜ 2 ⬜ 3 ⬜ 4

- membre inférieur droit

absent ⬜ présent ⬜ 0 ⬜ 1 ⬜ 2 ⬜ 3 ⬜ 4

- membre inférieur gauche

absent ⬜ présent ⬜ 0 ⬜ 1 ⬜ 2 ⬜ 3 ⬜ 4

**Rigidité** (le patient est maximalement détendu et allongé ou assis)

0 : pas de signe de rigidité

1 : au repos pas de rigidité, manœuvre de Froment positive (lever le bras controlatéral)

2 : minime rigidité présente même au repos complet (tester avec appui sur

articulation)

3 : rigidité légère (l’examinateur se rend tout de suite compte de la rigidité, mais l’amplitude du mouvement passif n’est point limitée et la rigidité est surmontée avec très peu de force)

4 : rigidité modérée (l’amplitude du mouvement passif n’est pas limitée mais la rigidité est surmontée seulement avec quelque force)

5 : rigidité prononcée (l’amplitude du mouvement passif peut être un peu limitée, la rigidité est surmontée seulement avec plus de force, équivalent à l’UPDRS III)

D : avec roue dentée

X : ne se détend pas suffisamment

- cou (flexion/extension et rotation) ⬜ 0 ⬜ 1 ⬜ 2 ⬜ 3 ⬜ 4 ⬜ 5

⬜ D ⬜ X

- axe (l’examinateur bouge les épaules du patient assis ou debout en rotation) ⬜ 0 ⬜ 1 ⬜ 2 ⬜ 3 ⬜ 4 ⬜ 5

⬜ D ⬜ X

- épaule D (rotation et élévation séparément)

⬜ 0 ⬜ 1 ⬜ 2 ⬜ 3 ⬜ 4 ⬜ 5

⬜ D ⬜ X

- épaule G (rotation et élévation séparément)

⬜ 0 ⬜ 1 ⬜ 2 ⬜ 3 ⬜ 4 ⬜ 5

⬜ D ⬜ X

- coude D (flexion/extension et pronation/supination séparément)

⬜ 0 ⬜ 1 ⬜ 2 ⬜ 3 ⬜ 4 ⬜ 5

⬜ D ⬜ X

- coude G (flexion/extension et pronation/supination séparément)

⬜ 0 ⬜ 1 ⬜ 2 ⬜ 3 ⬜ 4 ⬜ 5

⬜ D ⬜ X

- poignet D ⬜ 0 ⬜ 1 ⬜ 2 ⬜ 3 ⬜ 4 ⬜ 5

⬜ D ⬜ X

- poignet G ⬜ 0 ⬜ 1 ⬜ 2 ⬜ 3 ⬜ 4 ⬜ 5

⬜ D ⬜ X

- hanche D (rotation) ⬜ 0 ⬜ 1 ⬜ 2 ⬜ 3 ⬜ 4 ⬜ 5

⬜ D ⬜ X

- hanche G (rotation) ⬜ 0 ⬜ 1 ⬜ 2 ⬜ 3 ⬜ 4 ⬜ 5

⬜ D ⬜ X

- genou D ⬜ 0 ⬜ 1 ⬜ 2 ⬜ 3 ⬜ 4 ⬜ 5

⬜ D ⬜ X

- genou G ⬜ 0 ⬜ 1 ⬜ 2 ⬜ 3 ⬜ 4 ⬜ 5

⬜ D ⬜ X

- cheville D ⬜ 0 ⬜ 1 ⬜ 2 ⬜ 3 ⬜ 4 ⬜ 5

⬜ D ⬜ X

- cheville G ⬜ 0 ⬜ 1 ⬜ 2 ⬜ 3 ⬜ 4 ⬜ 5

⬜ D ⬜ X

⬜ D ⬜ X

- épaule D (rotation et élévation séparément)

⬜ 0 ⬜ 1 ⬜ 2 ⬜ 3 ⬜ 4 ⬜ 5

⬜ D ⬜ X

- épaule G (rotation et élévation séparément)

⬜ 0 ⬜ 1 ⬜ 2 ⬜ 3 ⬜ 4 ⬜ 5

⬜ D ⬜ X

- coude D (flexion/extension et pronation/supination séparément)

⬜ 0 ⬜ 1 ⬜ 2 ⬜ 3 ⬜ 4 ⬜ 5

⬜ D ⬜ X

- coude G (flexion/extension et pronation/supination séparément)

⬜ 0 ⬜ 1 ⬜ 2 ⬜ 3 ⬜ 4 ⬜ 5

⬜ D ⬜ X

- poignet D ⬜ 0 ⬜ 1 ⬜ 2 ⬜ 3 ⬜ 4 ⬜ 5

⬜ D ⬜ X

- poignet G ⬜ 0 ⬜ 1 ⬜ 2 ⬜ 3 ⬜ 4 ⬜ 5

⬜ D ⬜ X

- hanche D (rotation) ⬜ 0 ⬜ 1 ⬜ 2 ⬜ 3 ⬜ 4 ⬜ 5

⬜ D ⬜ X

- hanche G (rotation) ⬜ 0 ⬜ 1 ⬜ 2 ⬜ 3 ⬜ 4 ⬜ 5

⬜ D ⬜ X

- genou D ⬜ 0 ⬜ 1 ⬜ 2 ⬜ 3 ⬜ 4 ⬜ 5

⬜ D ⬜ X

- genou G ⬜ 0 ⬜ 1 ⬜ 2 ⬜ 3 ⬜ 4 ⬜ 5

⬜ D ⬜ X

- cheville D ⬜ 0 ⬜ 1 ⬜ 2 ⬜ 3 ⬜ 4 ⬜ 5

⬜ D ⬜ X

- cheville G ⬜ 0 ⬜ 1 ⬜ 2 ⬜ 3 ⬜ 4 ⬜ 5

⬜ D ⬜ X

# Annexe 9 : QUESTIONNAIRE DE QUALITE DE VIE PDQ 39 ET SYSTEME DE

# COTATION POUR LA MALDIE DE PARKINSON

A cause de votre maladie de parkinson, combien de fois avez-vous vécu l’une quelconque des situations suivantes, au cours du mois précédent ?

1 – Avez-vous eu des difficultés dans la pratique de vos loisirs ?

Jamais

Rarement

Parfois

Souvent

Toujours ou totalement incapable

2 – Avez-vous eu des difficultés à vous occuper de votre maison par exemple bricolage, ménage,

cuisine ?

Jamais

Rarement

Parfois

Souvent

Toujours ou totalement incapable

3 – Avez-vous eu des difficultés à porter des sacs de provisions ?

Jamais

Rarement

Parfois

Souvent

Toujours ou totalement incapable

4 – Avez-vous eu des problèmes pour faire 1 kilomètre à pied ?

Jamais

Rarement

Parfois

Souvent

Toujours ou totalement incapable

5 – Avez-vous eu des problèmes pour faire 100 mètres à pied ?

Jamais

Rarement

Parfois

Souvent

Toujours ou totalement incapable

6 – Avez-vous eu des problèmes à vous déplacer chez vous, aussi aisément que vous l’auriez

souhaité ?

Jamais

Rarement

Parfois

Souvent

Toujours ou totalement incapable

7 – Avez-vous eu des difficultés à vous déplacer dans les lieux publics ?

Jamais

Rarement

Parfois

Souvent

Toujours ou totalement incapable

*Veuillez vérifier que vous avez coché une case pour chaque question avant de passer à la page suivante.*

A cause de votre maladie de parkinson, combien de fois avez-vous vécu l’une quelconque des situations suivantes, au cours du mois précédent ?

8 – Avez-vous eu besoin de quelqu’un pour vous accompagner lors de vos sorties?

Jamais

Rarement

Parfois

Souvent

Toujours ou totalement incapable

9 – Avez-vous eu peur ou êtes-vous senti(e) inquiet (te) à l’idée de tomber en public ?

Jamais

Rarement

Parfois

Souvent

Toujours ou totalement incapable

10 – Avez-vous été confiné(e) chez vous plus que vous ne l’auriez souhaité ?

Jamais

Rarement

Parfois

Souvent

Toujours ou totalement incapable

11 – Avez-vous eu des difficultés pour vous laver ?

Jamais

Rarement

Parfois

Souvent

Toujours ou totalement incapable

12 – Avez-vous eu des difficultés pour vous habiller ?

Jamais

Rarement

Parfois

Souvent

Toujours ou totalement incapable

13 – Avez-vous eu des problèmes pour boutonner vos vêtements ou pour lacer vos chaussures ?

Jamais

Rarement

Parfois

Souvent

Toujours ou totalement incapable

14 – Avez-vous eu des problèmes pour écrire lisiblement?

Jamais

Rarement

Parfois

Souvent

Toujours ou totalement incapable

*Veuillez vérifier que vous avez coché une case pour chaque question avant de passer à la page suivante.*

A cause de votre maladie de parkinson, combien de fois avez-vous vécu l’une quelconque des situations suivantes, au cours du mois précédent ?

15 – Avez-vous eu des difficultés pour couper la nourriture ?

Jamais

Rarement

Parfois

Souvent

Toujours ou totalement incapable

16 - Avez-vous eu des difficultés pour tenir un verre sans le renverser ?

Jamais

Rarement

Parfois

Souvent

Toujours ou totalement incapable

17 – Vous êtes-vous senti(e) déprimé(e) ?

Jamais

Rarement

Parfois

Souvent

Toujours ou totalement incapable

18 – Vous êtes-vous senti(e) isolé(e) et seul(e)?

Jamais

Rarement

Parfois

Souvent

Toujours ou totalement incapable

19 – Vous êtes-vous senti(e) au bord des larmes ou avez-vous pleuré ?

Jamais

Rarement

Parfois

Souvent

Toujours ou totalement incapable

20 – Avez-vous ressenti de la colère ou de l’amertume ?

Jamais

Rarement

Parfois

Souvent

Toujours ou totalement incapable

21 – Vous êtes-vous senti (e) anxieux (se) ?

Jamais

Rarement

Parfois

Souvent

Toujours ou totalement incapable

*Veuillez vérifier que vous avez coché une case pour chaque question avant de passer à la page suivante.*

A cause de votre maladie de parkinson, combien de fois avez-vous vécu l’une quelconque des situations suivantes, au cours du mois précédent ?

22 - Vous êtes-vous senti (e) inquiet (te) pour votre avenir ?

Jamais

Rarement

Parfois

Souvent

Toujours ou totalement incapable

23 – Avez-vous ressenti le besoin de dissimuler aux autres votre maladie de Parkinson?

Jamais

Rarement

Parfois

Souvent

Toujours ou totalement incapable

24 – Avez-vous évité des situations où vous deviez manger ou boire en public?

Jamais

Rarement

Parfois

Souvent

Toujours ou totalement incapable

25 - Vous êtes-vous senti(e) gêné(e) en public à cause de votre maladie de Parkinson ?

Jamais

Rarement

Parfois

Souvent

Toujours ou totalement incapable

26 – Vous êtes-vous senti(e) inquiet(te) des réactions des autres à votre égard ?

Jamais

Rarement

Parfois

Souvent

Toujours ou totalement incapable

27 – Avez-vous eu des problèmes dans vos relations avec vos proches ?

Jamais

Rarement

Parfois

Souvent

Toujours ou totalement incapable

28 – Avez-vous manqué du soutien, dont vous aviez besoin, de la part de votre époux (se)

ou conjoint (e) ?

Jamais

Rarement

Parfois

Souvent

Toujours ou totalement incapable

*Veuillez vérifier que vous avez coché une case pour chaque question avant de passer à la page suivante.*

A cause de votre maladie de parkinson, combien de fois avez-vous vécu l’une quelconque des situations suivantes, au cours du mois précédent ?

29 – Avez-vous manqué du soutien dont vous aviez besoin, de la part de votre famille ou de vos amis

proches?

Jamais

Rarement

Parfois

Souvent

Toujours ou totalement incapable

30 – Vous êtes-vous endormi(e) dans la journée de façon inattendue ?

Jamais

Rarement

Parfois

Souvent

Toujours ou totalement incapable

31 – Avez-vous eu des problèmes de concentration, par exemple en lisant ou en regardant la

télévision ?

Jamais

Rarement

Parfois

Souvent

Toujours ou totalement incapable

32 – Avez-vous senti que votre mémoire était mauvaise ?

Jamais

Rarement

Parfois

Souvent

Toujours ou totalement incapable

33 – Avez-vous fait des mauvais rêves, ou eu des hallucinations ?

Jamais

Rarement

Parfois

Souvent

Toujours ou totalement incapable

34 – Avez-vous eu des difficultés pour parler ?

Jamais

Rarement

Parfois

Souvent

Toujours ou totalement incapable

35 – Vous êtes-vous senti(e) incapable de communiquer normalement avec les autres ?

Jamais

Rarement

Parfois

Souvent

Toujours ou totalement incapable

*Veuillez vérifier que vous avez coché une case pour chaque question avant de passer à la page suivante.*

A cause de votre maladie de parkinson, combien de fois avez-vous vécu l’une quelconque des situations suivantes, au cours du mois précédent ?

36 – Vous êtes-vous senti(e) ignoré(e) par les autres ?

Jamais

Rarement

Parfois

Souvent

Toujours ou totalement incapable

37 – Avez-vous eu des crampes ou des spasmes musculaires douloureux ?

Jamais

Rarement

Parfois

Souvent

Toujours ou totalement incapable

38 – Avez-vous eu mal ou avez-vous eu des douleurs dans les articulations ou dans le corps ?

Jamais

Rarement

Parfois

Souvent

Toujours ou totalement incapable

39 – Avez-vous eu la sensation désagréable de chaud et de froid ?

Jamais

Rarement

Parfois

Souvent

Toujours ou totalement incapable

Annexe 10 : BREF


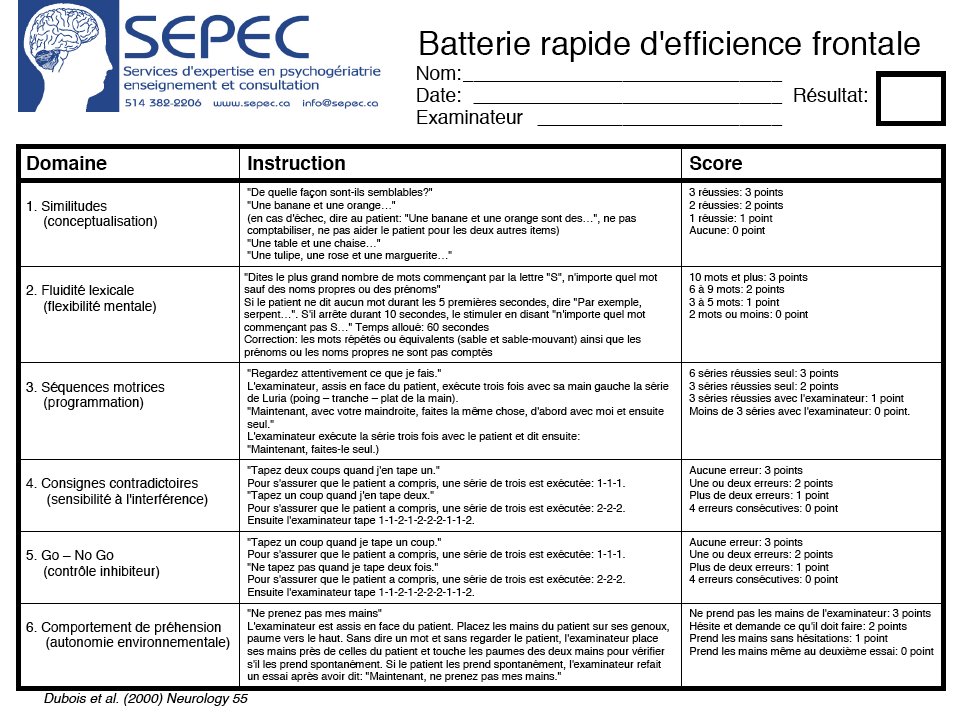

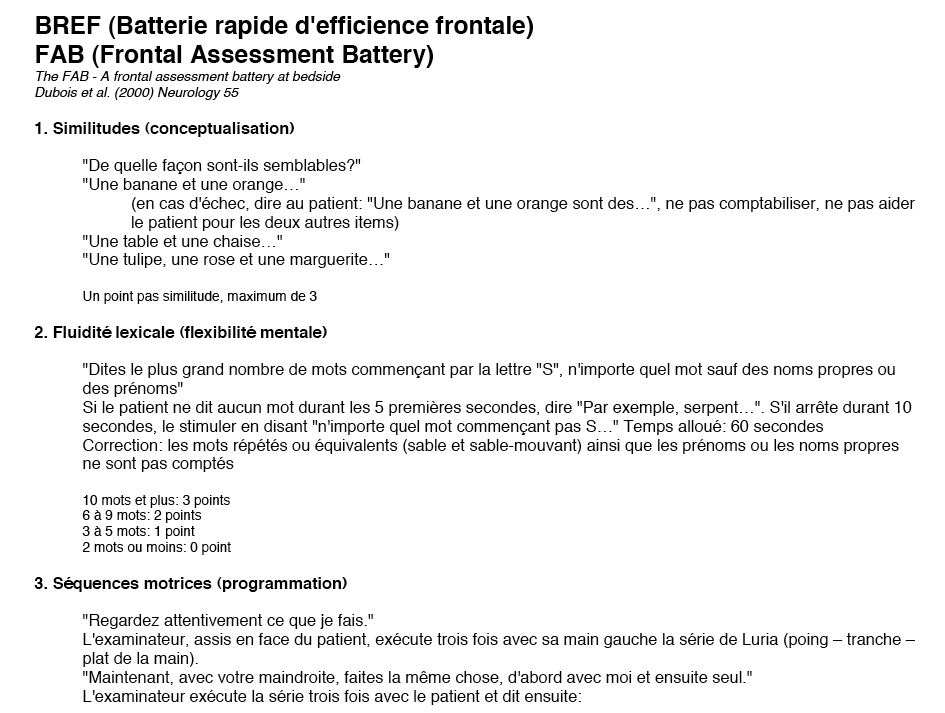

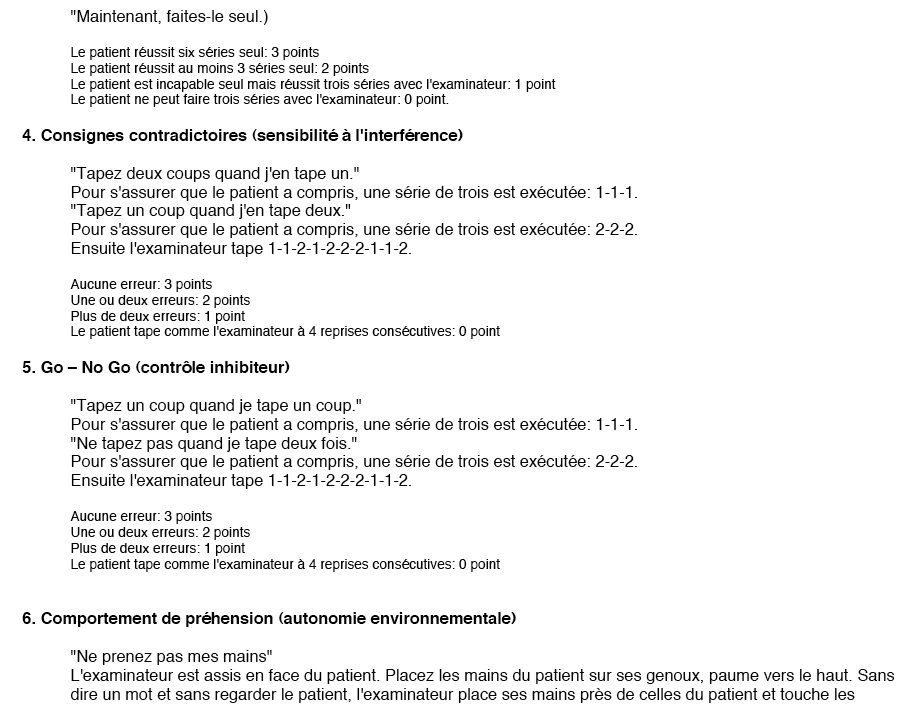

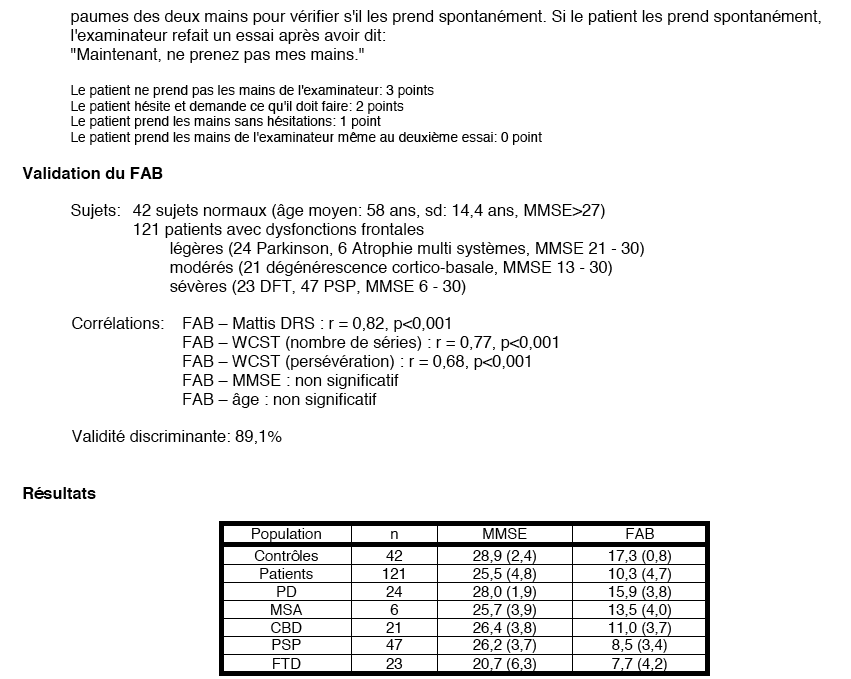

Supplement: S1 Protocol — (DOC) [file pone.0158235.s003.doc]
